# Targeting cancer hallmarks using resveratrol: isolation, formulation, and mechanisms of action

**DOI:** 10.3389/fnut.2026.1747820

**Published:** 2026-02-09

**Authors:** Wamidh H. Talib, Hadeel Shaher Al Junaidi, Rawan W. Hadi, Tassneem H. Ashour, Islam Z. Zabout, Suha M. Sabri, Heba K. Alshaeri, Douglas Law, Márta Hock, Viktória Prémusz

**Affiliations:** 1 Faculty of Allied Medical Sciences, Applied Science Private University, Amman, Jordan; 2 Faculty of Allied Medical Sciences, Department of Clinical Nutrition and Dietetics, Applied Science Private University, Amman, Jordan; 3 Faculty of Pharmacy, Al-Azhar University of Gaza, Gaza, Palestine; 4 Faculty of Medicine, Department of Pharmacology, King Abdulaziz University, Rabigh, Saudi Arabia; 5 Faculty of Health and Life Sciences, Inti International University, Nilai, Malaysia; 6 Faculty of Health Sciences, Institute of Physiotherapy and Sports Science, University of Pécs, Pécs, Hungary; 7 Physical Activity Research Group, János Szentágothai Research Center, University of Pécs, Pécs, Hungary; 8 Faculty of Health Sciences, Institute of Physiotherapy and Sports Science, University of Pécs, Pécs, Hungary; 9 Physical Activity Research Group, János Szentágothai Research Center, National Laboratory on Human Reproduction, University of Pécs, Pécs, Hungary

**Keywords:** angiogenesis, anticancer, cancer hallmarks, immune evasion, metastasis, resveratrol

## Abstract

Natural products, particularly medicinal plants, play a crucial role in combating cancer and developing new treatments due to their unique properties, such as diverse chemical structures, low toxicity, and the ability to target various types of cancer. They offer a promising approach for the treatment and prevention of certain cancers. Resveratrol, a non-flavonoid polyphenolic compound found in several plants, has demonstrated beneficial effects on various diseases, including cancer, as evidenced by numerous studies. It may also help address multiple cancers by influencing their growth and metastasis. One proposed mechanism is that resveratrol reduces reactive oxygen species (ROS) and lipid peroxidation by activating the JNK/c-JUN signaling pathway, which enhances the expression of antioxidant enzymes. Additionally, resveratrol has been shown to regulate cell proliferation by suppressing the PI3K/Akt pathway and activating the SIRT1 pathway. Other mechanisms include the inhibition of NF-κB activation and the downregulation of downstream proteins such as MMP-9, CXCR4, and FAK, which are known to facilitate metastasis. Furthermore, resveratrol inhibits Akt phosphorylation, and inhibition of PI3K or mTOR mimics its effects on glucose uptake. This review discusses these mechanisms, emphasizing the anticancer properties of resveratrol and its role in various aspects of cancer, supported by research on the compound’s formulation and clinical studies involving this natural agent.

## 1 Introduction

Cancer is one of the most lethal diseases, and the second leading cause of death after cardiovascular diseases (1). Due to the wide range of side effects associated with existing cancer treatments, which often lack specificity, there is a pressing need to focus on developing novel preventative and therapeutic approaches. Natural products represent a promising source for the development of efficient anticancer medications, owing to their diverse chemical structures, low toxicity, and ability to target multiple types of cancer (2).

Resveratrol, a powerful bioactive compound found in grapes, is one such substance. Numerous studies have reported its anticancer potential across various tumor types. Additionally, it possesses well-documented antioxidant properties (3). Research has demonstrated that resveratrol exhibits a range of anti-aging, cardioprotective, anti-inflammatory, and anticancer effects in both animals and humans (4). According to the literature, it may target several hallmarks of cancer, which are the fundamental biological processes and characteristics that contribute to the initiation and progression of cancer (5). Furthermore, comparative studies between resveratrol and other polyphenols have shown that resveratrol is the most potent Notch activator, a pathway that plays an important role in angiogenesis and tumor progression (6). Compared to flavonoids, resveratrol has also been found to be more potent in protecting against copper-catalyzed oxidation (7).

The biological capacities acquired during the multi-step progression of human malignancies are known as the hallmarks of cancer. These include maintaining proliferative signaling, evading growth suppressors, preventing apoptosis, enabling replicative immortality, inducing invasion and metastasis, altering energy metabolism, promoting angiogenesis, and evading immune destruction. One notable phytoalexin is resveratrol, which is characterized by its low molecular weight. More than 70 plant species produce resveratrol in response to stressful conditions as a defense mechanism against toxins, parasites, fungal infections, and ultraviolet (UV) radiation. Due to its ability to target multiple pathways, resveratrol is effective in inhibiting cancer growth (8). This review aims to provide an overview of the antitumor effects of resveratrol against various types of tumors, organized according to the hallmarks of cancer. It covers the definition, sources, physical and chemical properties, extraction and purification techniques, formulations for cancer treatment, and the most significant human clinical trials involving resveratrol.

## 2 Cancer hallmarks as therapeutic targets

**Figure 1:**
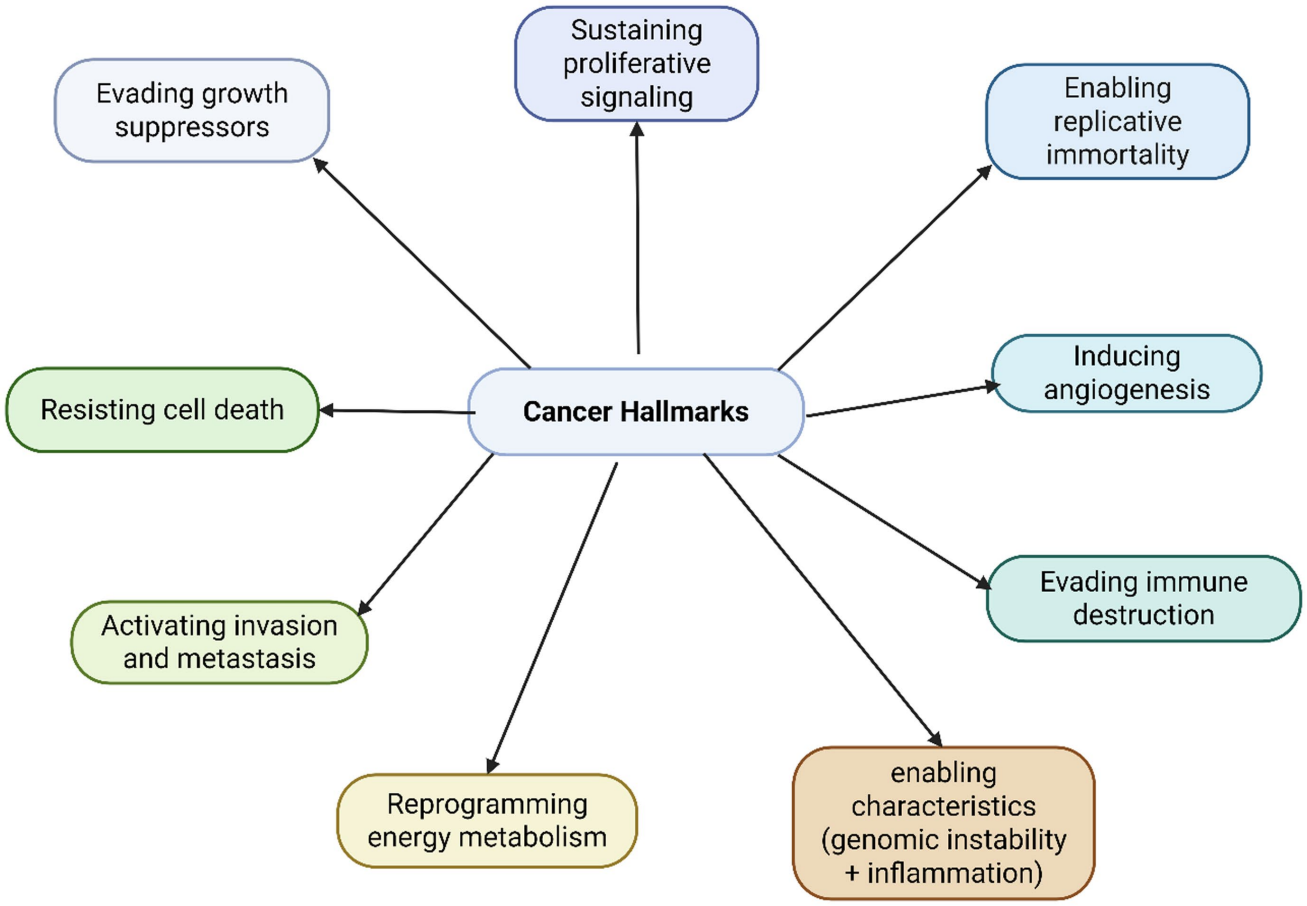
Cancer hallmarks.

Hallmarks of cancer are traits or functions that disrupt the body’s normally well-regulated cell and organ activities (Figure 1). They allow tumors to perform actions that normal cells cannot. Each hallmark and its related facilitators have been targeted in therapeutic development. Although these treatments often produce remarkable results, they tend to be short-lasting. Because cancer cells are highly adaptable, treatment-resistant forms can quickly develop (9).

### 2.1 Maintenance of proliferation

The normal structure and function of tissues are regulated by growth-promoting signals that guide cell cycle progression. By manipulating these signals, cancer cells can influence their fate. Growth factors that bind to cell-surface receptors—typically containing intracellular tyrosine kinase domains—play a crucial role in transmitting these enabling signals. These signals subsequently activate branching intracellular signaling pathways that regulate both cell development and progression through the cell cycle. Often, these signals also affect other cellular characteristics, such as survival and metabolic processes related to energy production (10).

There are several additional mechanisms by which cancer cells acquire the ability to sustain proliferative signaling. For instance, they may produce their own growth factor ligands and subsequently express the corresponding receptors, thereby initiating autocrine proliferative processes. Conversely, normal cells within the supportive tumor-associated stroma may receive signals from cancer cells that stimulate their activity. In turn, these normal cells can supply cancer cells with various growth factors. Furthermore, an increase in the number of receptor proteins on the cancer cell surface can lead to dysregulated receptor signaling (11).

Cancer involves cells that divide and proliferate uncontrollably. Growth factor signaling is disrupted, and cancer cells produce chemicals that further promote their proliferation (12).

### 2.2 Avoiding growth suppressors

Cancer cells must evade robust mechanisms that inhibit cell proliferation, many of which depend on the activity of tumor suppressor genes. The RB (retinoblastoma protein) and TP53 (tumor protein P53) are encoded by two essential tumor suppressor genes. The RB protein integrates signals from various intracellular and extracellular sources. Consequently, cancer cells with dysfunction in the RB pathway lose a crucial gatekeeper of cell-cycle progression, allowing unchecked cell proliferation. Multiple lines of evidence indicate that both traditional proliferation suppressors—TP53 and RB—function as part of a broader network designed to provide functional redundancy, despite their critical roles in regulating cell proliferation (13).

### 2.3 Preventing cell death

Apoptosis, or programmed cell death, is a natural defense mechanism against the development of cancer. It involves upstream regulators and downstream effector components. Over the past 20 years, functional studies have demonstrated that apoptosis acts as a critical barrier to cancer progression. The apoptotic machinery comprises two primary regulatory pathways: the extrinsic apoptotic pathway, which involves the Fas ligand/Fas receptor and transmits signals that induce extracellular death, and the intrinsic pathway, which senses and integrates various intracellular signals. Each pathway activates specific initiator caspases (caspase-8 for the extrinsic pathway and caspase-9 for the intrinsic pathway), which then trigger a cascade of proteolytic events involving effector caspases (14). These effector caspases execute the final phase of apoptosis, during which the cell is systematically dismantled and ultimately engulfed by neighboring cells and specialized phagocytes. Apoptosis is typically initiated in cells damaged by trauma, radiation, or oxidative stress—factors that can cause DNA alterations potentially leading to cancer. However, cancer cells often evade this protective mechanism to survive and proliferate. The p53 gene, a crucial protein in the apoptotic pathway often called the “Guardian of the Genome,” is mutated in nearly half of all malignancies (15).

### 2.4 Facilitating immortality through replication

It is widely recognized that for cancer cells to develop macroscopic tumors, they must possess an unlimited capacity for replication. In contrast, most of the body’s normal cell lineages can undergo only a finite number of cell divisions and proliferation cycles. This limitation is associated with two distinct barriers to proliferation: senescence, which is typically irreversible and involves entering a viable but non-proliferative state, and crisis, which results in cell death. In cultured cells, senescence is initially triggered by repeated rounds of cell division. Cells that overcome this barrier then enter the crisis phase, during which the vast majority of the cell population dies (16).

Senescence and crisis/apoptosis are two significant barriers to cellular growth and are considered essential anticancer defense mechanisms inherent in our cells. One or both of these processes halt most early-stage neoplasms once they have exhausted their capacity for replicative divisions. The ends of normal cells’ chromosomes, known as telomeres, progressively shorten and deteriorate as cells age, signaling that the cell is approaching death. This shortening is a hallmark of cellular aging. However, cancer cells have developed mechanisms to maintain their telomere length above a critical threshold, enabling them to divide indefinitely and achieve cellular immortality. Among the strategies cancer cells use to prevent telomere degradation, the most common is the activation of the enzyme telomerase, which protects chromosome ends. This extended lifespan is a key factor that allows cancer cells to proliferate and metastasize throughout the body (17).

### 2.5 Tumor-induced inflammation

The immune system’s attempts to combat cancer cells can sometimes provoke inflammatory responses that promote the growth of these cells. Immune cells may mistakenly recognize tumors as “non-healing wounds,” inadvertently supporting their survival, self-repair, and aggressive proliferation (18).

Most tumors trigger a tumor-promoting inflammatory response, which is recognized as a hallmark of cancer. Inflammation plays a crucial role at every stage of cancer development. The inflammatory microenvironment can increase mutations and genetic instability through the production of reactive oxygen species (ROS) and reactive nitrogen intermediates or via cytokines that stimulate ROS production (19). Inflammatory cytokines also induce activation-induced cytidine deaminase and inhibit p53, thereby increasing genetic instability and mutations. Moreover, inflammation enhances stem cell expansion and induces genes that promote cell proliferation and survival (20). Tumor-associated macrophages produce vascular endothelial growth factor (VEGF), promoting neoangiogenesis, while inflammatory myeloid cells and cancer cells produce transforming growth factor *β* (TGF-β), which facilitates epithelial-mesenchymal transition (EMT) and metastasis (19). Inflammatory cells also release proteases that facilitate the proteolysis required for invasion. Furthermore, inflammation upregulates adhesion molecules, enhancing the attachment of metastatic cells to target organs (20).

### 2.6 Genomic mutations and instability

When the genetic stability of cancer cells is compromised, their DNA becomes damaged. These cells evade the body’s mechanisms that maintain genomic integrity, resulting in an increased mutation rate. This leads to alterations, rearrangements, or damage to essential proteins that normally protect our DNA. Many cancer cells exhibit inherent genomic instability, causing significant variation both within individual tumors and among different tumors (21).

Genome instability is thought to result from defects in cellular caretaker systems. Dysfunctions in specific components of these systems have been proposed to explain the increased mutability observed in cancer cells. Among these components, the p53 tumor suppressor protein is the most prominent, as it responds to DNA damage by inducing either cell cycle arrest to allow DNA repair or apoptosis if the damage is excessive. However, the function of the p53 DNA damage signaling pathway is lost in most human cancers (22). Additionally, numerous other genes involved in sensing and repairing DNA damage or ensuring correct chromosomal segregation during mitosis are frequently lost in various cancers (23).

### 2.7 Activating angiogenesis

Similar to normal tissues, tumors require nutrients and oxygen for survival, as well as a mechanism to eliminate metabolic waste and carbon dioxide. The formation of new blood vessels, known as angiogenesis, fulfills these needs for tumors. As tumors progress, an “angiogenic switch” is typically activated and remains active, leading to the continuous development of new blood vessels from normally quiescent vasculature, thereby supporting tumor growth (24). Some regulators of angiogenesis are signaling proteins that interact with either stimulatory or inhibitory receptors on the surface of vascular endothelial cells. Vascular endothelial growth factor-A (VEGF-A) and thrombospondin-1 (TSP-1) are well-known examples of angiogenesis inducers and inhibitors, respectively. The blood vessels formed in tumors due to persistently activated angiogenesis and an imbalanced mix of proangiogenic signals are often abnormal: tumor-associated blood vessels exhibit early capillary growth, excessive and twisted branching, distorted and enlarged structures, irregular blood flow, microbleeding, leakage, and abnormal rates of endothelial cell proliferation and death (25).

### 2.8 Initiating invasion and metastasis

The immune system eliminates abnormal or damaged cells before they can develop into cancerous tumors. However, cancer cells can adapt to evade detection and suppress immune responses. The complex process of invasion and metastasis is typically described as a series of distinct stages, often referred to as the invasion-metastasis cascade (26). This model outlines a sequence of biological changes in cells, beginning with local invasion, followed by cancer cells entering nearby blood and lymphatic vessels (intravasation), traveling through these systems, and ultimately escaping into surrounding tissues (extravasation) (26). This progression leads to the formation of small clusters of cancer cells (micrometastases), which can eventually grow into larger tumors, a process known as “colonization.” Cancer cells can disseminate throughout the body, disrupting the normal function of tissues and organs, which is a major reason why cancer treatment is so challenging (27).

### 2.9 Altering cellular energy processes

Cells require energy to perform various functions, including nutrient absorption, responding to environmental changes, and maintaining homeostasis. Cancer cells utilize energy for their growth in a manner distinct from that of normal cells. Their continuous growth is fueled by “glucose fueling,” a process in which cancer cells modify their metabolism by activating transporters on their membranes to enhance the uptake and utilization of energy-rich glucose (28).

Under aerobic conditions, normal cells metabolize glucose by first converting it to pyruvate via glycolysis in the cytosol, followed by its complete oxidation to carbon dioxide in the mitochondria. When oxygen is limited, glycolysis is favored, and only a small portion of pyruvate enters the mitochondria. In contrast, cancer cells alter their glucose metabolism even in the presence of oxygen by relying primarily on glycolysis for energy production, a phenomenon known as “aerobic glycolysis” (9, 29).

Since glycolysis is approximately 18 times less efficient at generating ATP compared to mitochondrial oxidative phosphorylation, cancer cells compensate by increasing the expression of glucose transporters, particularly GLUT1, which enhances glucose uptake into the cytoplasm (29, 30).

### 2.10 Escaping immune destruction

Typically, the body’s vigilant immune system identifies and eliminates abnormal or damaged cells before they can develop into cancerous tumors. However, cancer cells can evolve mechanisms to evade detection and suppress the immune system components designed to target and destroy them (9).

Tumors evade immune destruction through multiple mechanisms. Transforming growth factor beta (TGF-*β*) plays a critical role by inhibiting T helper cell differentiation and suppressing antitumor immunity (31). Tumor-derived factors convert immature myeloid cells into myeloid-derived suppressor cells (MDSCs), which inhibit the antitumor immune response (32). Additionally, tumor exosomes can impair T cell function and induce the generation of monocytic MDSCs, further hindering tumor recognition by immune cells (33). The extracellular matrix (ECM) also impairs antigen-presenting cells and inhibits T cell activation, thereby suppressing T cell-mediated antitumor responses (34).

## 3 Resveratrol

### 3.1 Resveratrol’s chemical structure

Resveratrol (3,5,4-trihydroxystilbene) is a naturally occurring polyphenol with a stilbene structure, first isolated from the root of *Veratrum grandiflorum* by Takaoka in 1940 (35). It is the fundamental molecule of a class of compounds that includes polymers, such as viniferins, and glycosides, such as piceid. *Polygonum cuspidatum*, commonly known as Japanese knotweed, is a plant used for millennia in traditional Asian medicine to treat inflammation and various health conditions. It contains the highest natural concentrations of resveratrol (36).

**Figure 2:**
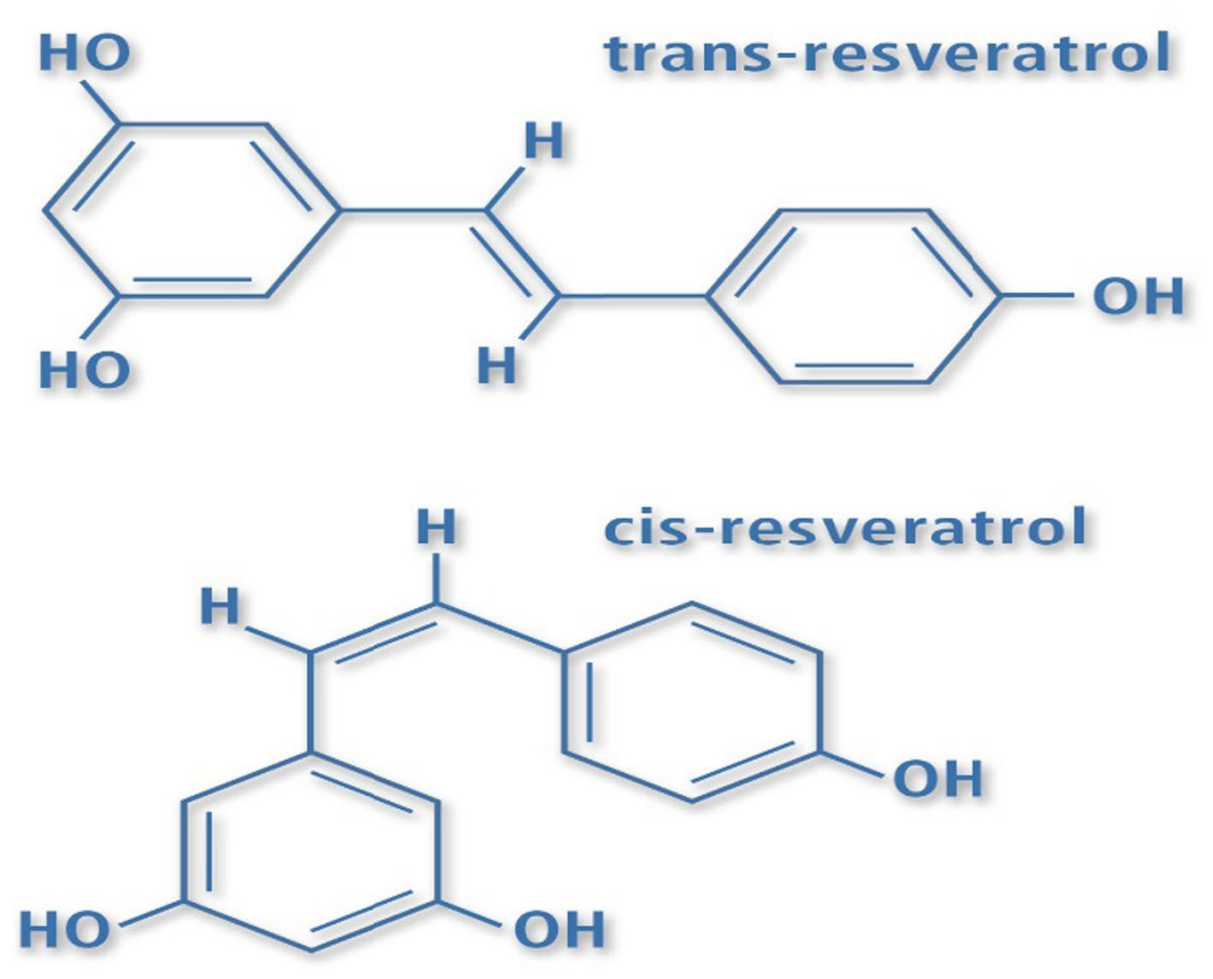
The structures of *cis*-resveratrol and *trans*-resveratrol.

Resveratrol consists of two aromatic rings linked by a methylene bridge and can exist in both cis and trans forms (Figure 2). The trans form, which is the more common and stable natural variant, can convert to the cis form when exposed to heat, ultraviolet light, or sunlight. These structures can bind to glucose, forming stilbene glycosides known as cis- and trans-piceid. In the digestive system, these glycosides can be hydrolyzed by the action of β-glucosidase, releasing resveratrol (37).

### 3.2 Physical and chemical properties of resveratrol

Resveratrol is a low molecular weight phyto-polyphenol (228 Da) that appears as an off-white powder with lipophilic properties. Its melting point ranges between 253 and 255 °C, and it has very low solubility in water. Trans-resveratrol can isomerize to the cis form through molecular breakdown when exposed to UV radiation (254 or 366 nm), elevated pH, or high temperatures. Due to its poor water solubility, resveratrol exhibits reduced concentration in the stratum corneum, which limits its ability to permeate the skin (38). Pre-formulation studies indicate that the stability of resveratrol is significantly affected by factors such as UV exposure, pH, and temperature, all of which are critical considerations during the formulation of trans-resveratrol (39).

### 3.3 Absorption

Resveratrol has low water solubility (<0.05 mg/mL), which adversely affects its absorption. It is absorbed through passive diffusion and can be rapidly metabolized by the gut microbiome and liver following oral intake. Three primary metabolites are produced: resveratrol-3-O-sulfate, resveratrol-4′-O-glucuronide, and resveratrol-3-O-glucuronide. The free form of resveratrol can bind to albumin and lipoproteins, such as low-density lipoprotein (LDL). These metabolic processes reduce the amount of resveratrol available for physiological functions (40, 41). Several methods have been found to enhance its solubility, including the use of organic solvents such as ethanol (50 mg/mL), acetylation of resveratrol, and the development of supplements to increase resveratrol intake (42).

### 3.4 Metabolism

**Figure 3:**
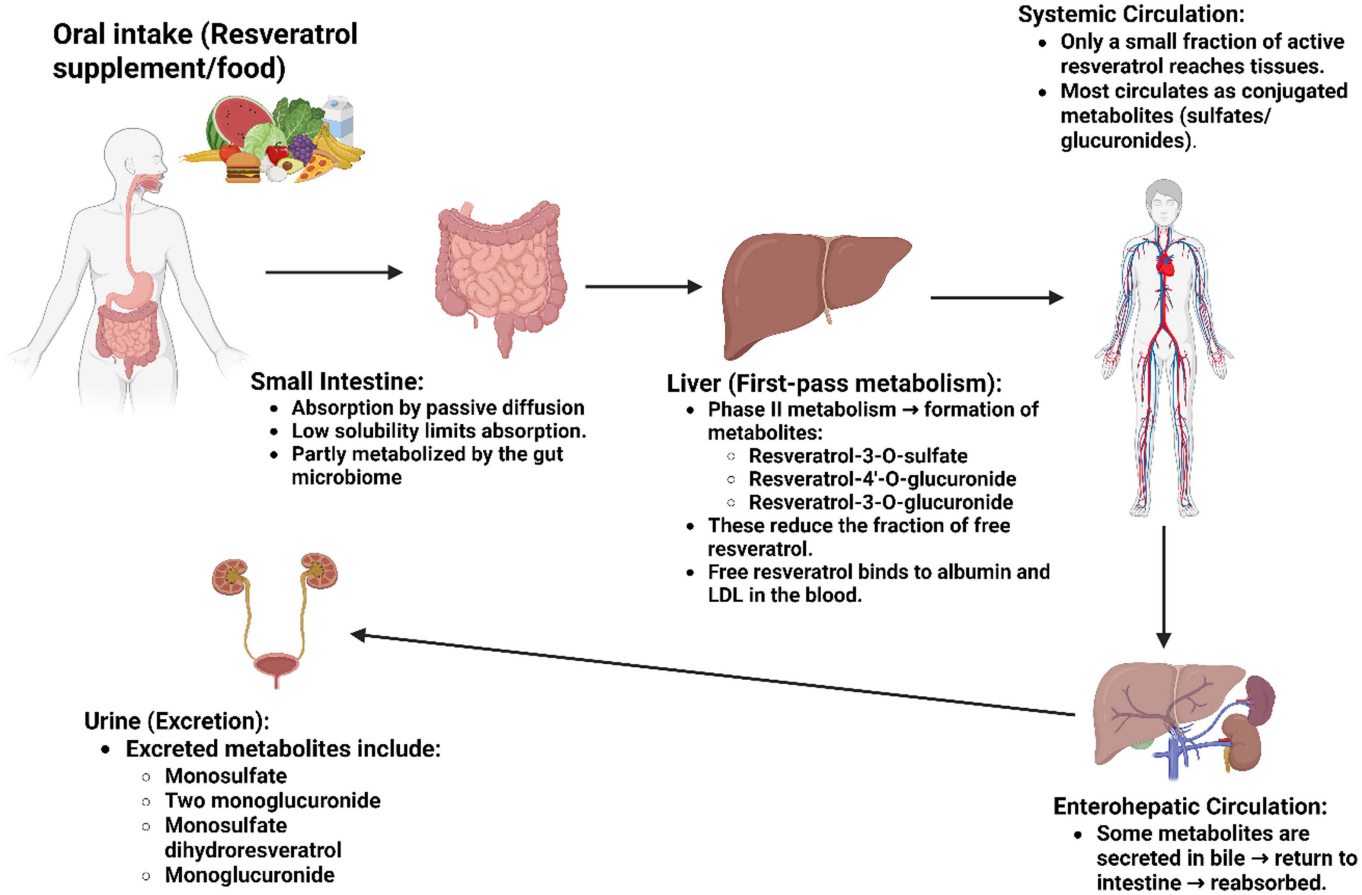
Absorption and metabolism of resveratrol.

Resveratrol is primarily metabolized in the liver through Phase II metabolic processes. It undergoes enterohepatic circulation via bile, which can lead to its reabsorption in the small intestine (5). Resveratrol is extensively metabolized, resulting in the formation of conjugated sulfates and glucuronides that still retain some biological activity (Figure 3). In urine, up to five different metabolites can be identified, including resveratrol monosulfate, two isomeric forms of resveratrol monoglucuronide, monosulfate dihydroresveratrol, and monoglucuronide. It is important to note that the types and quantities of these metabolites can vary among individuals due to inherent biological differences (43). Additionally, cis isomers are less stable than trans isomers; however, research suggests that both forms may exhibit distinct biological effects (44).

### 3.5 Bioavailability

Resveratrol exhibits strong biological effects *in vitro*. When taken orally, its lipophilic properties enhance absorption; however, the extent of absorption varies depending on the method of consumption and the type of food ingested (45). Its poor oral bioavailability limits clinical use, primarily due to low water solubility (46). Following oral administration, resveratrol is rapidly conjugated in enterocytes and hepatocytes via phase II metabolic pathways, mainly glucuronidation and sulfation, mediated by UDP-glucuronosyltransferases (UGTs) and sulfotransferases (SULTs). Consequently, free resveratrol levels in the bloodstream remain low, with conjugated metabolites constituting the majority of plasma concentrations (47). Efflux transporters such as P-glycoprotein may further reduce its intracellular availability (48). Despite high absorption, pharmacokinetic studies indicate that rapid metabolism and clearance result in a short systemic half-life and low bioavailability of the parent compound (49). These pharmacokinetic challenges highlight the need for bioactive derivatives or optimized delivery systems to enhance therapeutic efficacy (50). Nevertheless, resveratrol demonstrates *in vivo* efficacy due to the conversion of its metabolites—sulfates and glucuronides—back to resveratrol in target organs such as the liver. Moreover, resveratrol can be deconjugated in the small intestine and subsequently reabsorbed. The cis form of resveratrol exhibits lower bioavailability because it undergoes glucuronidation at a rate five to ten times faster than the trans form (51). A pharmacokinetic study involving repeated doses over two days indicated that tolerance is well established, plasma concentrations decrease over time, and bioavailability is greater when taken in the morning (52). Various strategies have been employed to enhance the bioavailability of resveratrol, including nanoencapsulation technology and other drug delivery systems. Recent studies suggest that bioavailability can be improved by combining resveratrol with additives that inhibit its metabolism *in vivo*, synthesizing analogs with enhanced bioavailability, and utilizing various nanotechnological methods. The incorporation of resveratrol into carriers such as *β*-cyclodextrin complexes, liposomes, and solid lipid nanoparticles has been documented, with nanoparticle encapsulation resulting in increased bioavailability compared to oral administration of resveratrol (42).

## 4 Resveratrol sources

Due to the health benefits associated with resveratrol, some individuals may consider increasing their intake of this compound. The primary dietary sources of resveratrol are grapes and wine, with concentrations varying based on factors such as grape variety, origin, ripeness, fermentation method, and duration. Typically, the concentration of resveratrol in red wine ranges from 1.98 to 7.13 mg/L, and it is generally higher in red wine compared to white wine (53, 54). Resveratrol is produced by over 70 plant species in response to environmental stress. Key dietary sources include peanuts, mulberries, blueberries, and strawberries (55). The amount of resveratrol present in plants is influenced by several factors, with weather conditions and the presence of fungi being particularly significant for grapevines (56). Additionally, due to its pharmacological properties, resveratrol supplements and health products have become widely available in the market (57).

Resveratrol supplements may offer a consistent and concentrated source of resveratrol, helping to mitigate the effects of seasonal fluctuations in fruit availability and enabling individuals to maintain adequate levels of this compound. Throughout the year, certain fruits exhibit high resveratrol content, with levels reaching up to 1,941 μg/100 g. For example, tangerines contain 1,061.43 μg/100 g, while peaches provide 461.6 μg/100 g, making both fruits excellent sources of resveratrol. In addition to tangerines and peaches, other fruits such as apples and grapefruits also contain resveratrol. Among these, tangerines have the highest resveratrol content, followed by peaches, oranges, grapefruits, and grapes. Apples and pears contain lower amounts, with 67 μg/100 g and 34.43 μg/100 g, respectively, while grapes and grapefruits contain 79.25 μg/100 g and 82 μg/100 g, respectively (58). This information suggests that a variety of fruits can serve as excellent sources of resveratrol, thereby contributing to a diverse daily diet.

The amount of resveratrol consumed can vary significantly throughout the year due to the seasonal availability of fruits, making it challenging for individuals to obtain sufficient quantities, especially when certain fruits are out of season. This variation can affect the health benefits associated with resveratrol. While nuts and sweet potatoes provide a year-round source of resveratrol, they contain relatively low levels of this compound and cannot fully substitute for seasonal fruits. Among nuts, walnuts have the highest resveratrol content at 1,585 μg/100 g, followed by peanuts at 74 μg/100 g. Potatoes also contain a notable amount of resveratrol at 952.4 μg/100 g. In contrast, coarse cereals and legumes have lower resveratrol levels, with oats, soybeans, mung beans, and red beans containing 56.5, 10.75, 2, and 0 μg/100 g, respectively (58). It is essential to monitor resveratrol intake in individuals’ daily diets, as excessive consumption may lead to side effects such as gastrointestinal discomfort, including diarrhea and nausea, typically occurring within an hour after ingestion (59) (Table 1).

**Table 1:** Resveratrol content in common foods.

Food Source	Resveratrol content (μg/100 g)	Rich plant part
Grapes	1,941 (up to 15,000 in some cultivars)	Skin, mainly, also leaves, and stems
Walnuts	1,585	Kernel
Potatoes	952.4	Skin and flesh
Tangerine	1,061.43	Peel mainly
Peach	461.6	Skin and flesh
Peanuts	74	Shell and seeds
Grapefruit	82	Peel
Apple	67	Peel
Oats	56.5	Whole grains
Pear	34.43	Peel
Soybeans	10.75	Seeds

## 5 Resveratrol isolation and purification

(E)-Resveratrol can be extracted from the bark of conifer trees, and the total phenolic content can be assessed using the Folin–Ciocalteu assay (60). Quantitative analysis of the compound is performed using HPLC-MS and HPLC-UV techniques. The purification process yields an extraction rate of 84% and a purity of 99% for (E)-resveratrol, effectively isolating the compound. One study indicates that (E)-resveratrol can be sourced from the bark of Black spruce (*Picea mariana*) for extraction and purification. In this study, 12.5 grams of dried, ground bark were extracted for 12 h with 125 mL of diethyl ether using a Soxhlet apparatus, followed by an additional 12-h extraction with another 125 mL of the same solvent. The two extracts were combined, and the total volume was reduced to 100 mL through rotary evaporation. The sample was then washed with a 5% (w/v) sodium bicarbonate solution (three times with 50 mL each). The organic phase (diethyl ether layer) was evaporated to near dryness and re-dissolved in methanol to achieve a final volume of 35.0 mL. A 10.0 mL aliquot of this sample was dried with 1 g of Sephadex LH-20 column material and loaded onto a column (10.5 mm ID × 140 mm H) prepared with the same material in dichloromethane. The sample was then washed three times with 50 mL of 5% (w/v) sodium bicarbonate solution. The organic phase (diethyl ether layer) was evaporated to near dryness and re-dissolved in methanol to a final volume of 35.0 mL. A 10.0 mL aliquot of this sample was dried with 1 g of Sephadex LH-20 column material and loaded onto a column (10.5 mm ID × 140 mm H) packed with the same material in dichloromethane. The sample was eluted with dichloromethane until 100 mL of fraction was collected, after which the mobile phase was switched to a 9:1 mixture of dichloromethane and methanol. Column fractions of 25 mL were collected and analyzed by HPLC until (E)-resveratrol was fully eluted. The fractions containing (E)-resveratrol were combined, concentrated by rotary evaporation, dissolved in methanol, and adjusted to a final volume (61). The Folin–Ciocalteu method is a widely employed technique for quantifying the total phenolic content in plant extracts. This method relies on the ability of phenolic compounds to reduce the Folin reagent under basic conditions, resulting in the formation of a colored complex (62).

In a separate study titled “Method for the Extraction and Purification of (E)-Resveratrol from Black Spruce Bark,” crude diethyl ether extracts of black spruce bark, after treatment with 5% sodium bicarbonate, showed a concentration of 332.9 ± 0.4 μg/g of (E)-resveratrol, as determined by HPLC analysis. The final purified sample contained 279.9 ± 4.9 μg/g of (E)-resveratrol, corresponding to an overall recovery rate of 84% (63).

Another study focuses on extracting resveratrol from *Polygonum cuspidatum* by leveraging the chemical properties of compounds in a 95% ethanol extract. This method involves reflux extraction, filtration, hydrolysis, liquid–liquid extraction, and elution (64). In conclusion, various techniques exist for extracting resveratrol, each producing different concentrations and yields of the compound.

## 6 Mechanism of resveratrol on each cancer Hallmark

### 6.1 Oxidative stress and DNA repair

Oxidative stress occurs when there is an imbalance between the production of reactive oxygen species (ROS) and the body’s antioxidant defenses. This imbalance arises either from excessive ROS production that exceeds the capacity of the antioxidant system or from a significant weakening of the antioxidant defenses. As a result, oxidative stress damages DNA and induces mutations in tumor suppressor genes, which are critical early events in cancer development (65).

Resveratrol is widely recognized for its role in the cellular defense system against oxidative stress and in maintaining DNA integrity. In a preclinical study conducted by Masoumeh Maleki et al. (66) using a rat model of colon cancer induced by 1,2-dimethylhydrazine (DMH), resveratrol demonstrated significant anti-proliferative effects by modulating oxidative stress and DNA repair mechanisms. The rats received resveratrol orally at a dosage of 8 mg/kg for 16 weeks, resulting in a substantial reduction in reactive oxygen species (ROS), lipid peroxidation, and protein carbonylation—key contributors to oxidative DNA damage and cancer progression. Mechanistically, resveratrol activated the JNK/c-JUN signaling pathway, which enhanced the expression of antioxidant enzymes, including superoxide dismutase (SOD), glutathione peroxidase (GPx), and catalase (CAT) (67). Moreover, resveratrol significantly upregulated base excision repair (BER) proteins, including 8-Oxoguanine DNA Glycosylase 1 (OGG1), X-Ray Repair Cross-Complementing Protein 1 (XRCC1), and Apurinic/Apyrimidinic Endonuclease 1 (APE1), indicating an improved DNA damage response and enhanced genomic stability. The activation of c-Jun N-terminal Kinase (JNK) signaling appears to function as a crucial upstream regulator, linking oxidative stress to the enhancement of DNA repair mechanisms. These findings highlight that resveratrol not only suppresses tumor progression but also supports redox homeostasis and DNA integrity by activating both antioxidant defenses and repair pathways (66). Consequently, it inhibits the growth of cancer cells, as discussed in the following section on the antiproliferative activity of resveratrol.

### 6.2 Cell proliferation and regulation

Cancer cells possess the capacity to induce cell division or proliferation.

The most common and well-established method of maintaining proliferative signaling is the mutational modification of cancer cells’ genes, which turns those genes into active cell proliferation drivers. In part, resveratrol research shows that resveratrol modulates important signaling pathways, transcriptional regulators, and cell cycle machinery to act as a complex regulator of cancer cell proliferation (68).

Building on evidence of its impact on signaling and transcriptional regulation, a previous study investigated resveratrol’s role in gastric cancer models. Hui Liu found that in gastric cancer cells lacking CDX2 (MKN7 and TMK1), resveratrol (Res) significantly reduced cell proliferation, migration, and invasion. The underlying mechanism involved Res, at concentrations of 50 and 100 μM, enhancing the expression of the tumor suppressors CDX2 and RUNX3, which subsequently inhibited the Wnt/*β*-catenin/TCF-4 signaling pathway—a critical pathway for cancer cell growth. Additionally, Res promoted apoptosis by downregulating Bcl-2 and upregulating Bax. Notably, Res counteracted the effects of LiCl, a Wnt activator, confirming its ability to inhibit β-catenin-mediated transcription. These findings demonstrate that Res suppresses tumor growth through the molecular regulation of key transcription factors and apoptotic proteins (69).

o further elucidate resveratrol’s anti-proliferative properties, Chun-Yin Huang conducted a study investigating its anticancer effects using *in vivo* breast cancer models, with a particular focus on sustained proliferation. Researchers administered resveratrol at doses ranging from 5 to 50 mg/kg of body weight daily to tumor-bearing mice over four weeks. The results demonstrated a dose-dependent inhibition of tumor growth, with the most significant effects observed at 50 mg/kg. Mechanistically, the study revealed that resveratrol exerts these antiproliferative effects through three key mechanisms: First, it suppresses the PI3K/Akt pathway, reducing cancer cell survival signals by approximately 40% compared to controls. Second, it upregulates the tumor suppressor proteins p53 and p21 by 2- to 3-fold, inducing cell cycle arrest at the G1/S checkpoint. Third, resveratrol treatment decreases the expression levels of cyclin D1 and CDK4/6 by 50–60%, effectively blocking cell cycle progression (68). While these findings strongly support resveratrol’s potential as an anticancer agent, the authors note important limitations. The effective concentrations used in this animal study (equivalent to approximately 400–4,000 mg daily for humans) far exceed typical dietary intake. Collectively, these findings highlight how resveratrol inhibits proliferative signaling by targeting stress-related pathways and transcriptional regulators (70).

The ability to transition cells from a proliferative to an apoptotic state suggests that resveratrol may enhance tumor sensitivity to tumor suppressors. In addition to directly targeting cancer cells, resveratrol has demonstrated protective effects in normal tissues by activating similar growth-suppressing pathways under stress conditions. For instance, a study on radiation-induced intestinal damage conducted by Haoren Qin found that resveratrol effectively protected normal epithelial cells through multiple growth-suppressing mechanisms (71).

At a concentration of 1 μM, resveratrol activated the SIRT1 pathway, which deacetylated and stabilized FOXO3a, a transcription factor that upregulates antioxidant enzymes such as SOD2 and CAT. This activation reduced oxidative stress and facilitated the proper regulation of cell survival. Furthermore, pro-apoptotic markers, including Bax and cleaved caspase-3, were expressed less frequently, and p53 acetylation was decreased by resveratrol. These changes prevented excessive apoptosis and regulated cell cycle arrest, mimicking the role of natural tumor suppressors. Inhibiting SIRT1 reversed these effects, confirming its essential role in resveratrol-mediated growth suppression. Thus, resveratrol supports anti-cancer therapy by reinforcing the body’s intrinsic tumor suppressor systems, particularly under stress conditions such as radiation exposure (71).

### 6.3 Cell apoptosis

Apoptosis is a form of programmed cell death essential for maintaining homeostasis and supporting development. Cancer is characterized by the dysregulation and evasion of apoptotic mechanisms, allowing tumor cells to survive. These cells employ various strategies to evade cell death, including the expression of apoptosis inhibitors, which contribute to resistance against cancer therapies (14).

Beyond its role in regulating proliferation and supporting normal cell survival, resveratrol is also essential for reactivating apoptosis, particularly in cancer cells that have developed resistance to conventional apoptotic pathways. Resveratrol induces apoptosis in cancer cells by activating both intrinsic (mitochondrial) and extrinsic apoptotic pathways. It promotes programmed cell death by facilitating the release of mitochondrial cytochrome c, increasing the Bax/Bcl-2 ratio, and activating caspase-9 and caspase-3. These effects are associated with the downregulation of survival pathways such as nuclear factor kappa B (NF-κB) and protein kinase B/mammalian target of rapamycin (Akt/mTOR), thereby enhancing the cells’ susceptibility to apoptosis (72). Additionally, resveratrol increases apoptosis in various tumor models by suppressing pro-survival signaling pathways (such as Akt and EGFR) and reactive oxygen species (ROS)-dependent mechanisms (73).

This study demonstrated a potent ability of resveratrol to induce apoptosis in acute lymphoblastic leukemia (ALL) cells, including those resistant to the CD95-mediated apoptotic pathway. Resveratrol concentrations ranging from 50 to 100 μM triggered nearly 80% apoptosis in various ALL cell lines, including pro-B, pre-B, and T-cell types, while sparing healthy peripheral blood mononuclear cells. Mechanistically, the compound initiated a mitochondrial-dependent pathway, characterized by an early and progressive loss of mitochondrial membrane potential (ΔΨm), followed by significant activation of caspase-9 within 48 to 72 h. Interestingly, blocking CD95 signaling or upregulating CD95L did not affect resveratrol’s action, confirming its independence from extrinsic apoptosis. The use of the pan-caspase inhibitor Z-VAD-FMK inhibited cell death but did not prevent the loss of mitochondrial membrane potential, indicating that caspase-9 activation occurs downstream of mitochondrial damage. These findings underscore resveratrol’s ability to circumvent resistance mechanisms in CD95-insensitive cancers and support its role in restoring apoptotic potential through intrinsic mitochondrial targeting. However, the high concentrations required *in vitro* pose a challenge for translating these results into clinical applications due to resveratrol’s limited bioavailability (74).

Supporting this mitochondrial-based mechanism, further research on melanoma cells has revealed that resveratrol can also promote apoptosis through a distinct, p53-independent pathway, highlighting its versatility in targeting resistant cancer types. A study by Hailong Zhao revealed that resveratrol triggers apoptosis through a mechanism that does not involve p53, specifically through the Erk/PKM2/Bcl-2 signaling pathway. Although initial treatment caused a 3.5-fold rise in p53 levels, experiments with p53-knockdown cells showed that apoptosis was mainly caused by the destabilization of Bcl-2, an important anti-apoptotic protein. Resveratrol (at concentrations of 50–200 μM) inhibited the phosphorylation of Erk1/2, which led to a decrease in PKM2, a vital enzyme in glycolysis that usually binds to and stabilizes Bcl-2. This disruption resulted in a 40% increase in the ubiquitination of Bcl-2, leading to its degradation. As a result, this process activated Bax and caused the release of cytochrome c from the mitochondria, thereby promoting apoptosis. Apoptosis plays a crucial role in cancer treatment. Notably, the overexpression of PKM2 reversed these effects, confirming its central role in the process. This study highlights a unique therapeutic mechanism through which resveratrol promotes apoptosis independently of p53, thereby expanding its potential utility against resistant cancer types (75). While resveratrol can restore apoptotic pathways in resistant cancer cells, it also interferes with another key hallmark of cancer—replicative immortality—by targeting telomerase activity and limiting uncontrolled cell division. Research has demonstrated that resveratrol inhibits cell proliferation and triggers apoptosis in human A431 epidermoid carcinoma cells in a dose-dependent manner by specifically targeting telomerase activity. At concentrations ranging from 0.1 to 0.3 mg/mL, resveratrol significantly reduced cell viability, accompanied by distinct signs of apoptosis, such as nuclear fragmentation and cytoplasmic condensation. Mechanistically, it suppressed telomerase activity by 14–30% and downregulated hTERT, the catalytic subunit of telomerase, as confirmed by TRAP-PCR-ELISA and Western blot analysis. These findings suggest that resveratrol inhibits hTERT transcription, thereby preventing telomerase activation, a crucial step in enabling cellular immortality. Replicative immortality in cancer cells is a significant concern. Although further *in vivo* studies are necessary, this evidence supports the potential of resveratrol as a cancer-fighting agent targeting one of the fundamental hallmarks of cancer (76).

### 6.4 Regulation of autophagy

In addition to inducing apoptosis, resveratrol modulates autophagy, a cellular process that can either promote cell survival or trigger cell death. Although various signaling pathways involved in resveratrol-induced autophagy have been proposed, the precise mechanisms by which resveratrol determines whether to initiate autophagy or apoptosis in different cancer cells remain unclear and require further investigation. mTOR, a serine/threonine kinase complex, is a central inhibitor of autophagy in mammalian cells and exists in two distinct complexes: mTOR complex 1 (mTORC1) and mTOR complex 2 (mTORC2) (77). Many cancer cells exhibit dysregulated mTOR signaling, making mTOR a promising target for cancer therapy. Resveratrol has been shown to enhance the interaction between mTOR and DEPTOR (DEP-domain containing mTOR-interacting protein), an endogenous inhibitor of mTOR (78). Additionally, activation of AMP-activated protein kinase (AMPK), an intracellular energy and metabolic sensor, plays a critical role in maintaining cellular energy homeostasis by phosphorylating various downstream targets (79). Raptor, a key component of the mTORC1 complex, is also phosphorylated by AMPK. Resveratrol can suppress mTORC1 activity either through AMPK activation or by directly inhibiting mTOR (80).

Moreover, resveratrol can induce mTOR-dependent autophagy and reduce cancer cell survival (81). As an activator of SIRT1, resveratrol has been reported to induce autophagy by inhibiting the Akt/mTOR pathway and activating p38-MAPK in non-small cell lung cancer (82). Resveratrol has been shown to participate in both pro-survival and pro-death cellular processes. The anticancer properties of resveratrol are likely due to its effects on multiple signaling pathways rather than a single pathway (83).

### 6.5 Telomerase inhibition

Telomerase activation is a crucial mechanism employed by cancer cells to maintain telomere length and achieve replicative immortality. Normal somatic cells exhibit low telomerase expression, whereas most cancer cells display high telomerase activity. This elevated telomerase activity in cancer primarily results from the upregulation of the human telomerase reverse transcriptase (hTERT) gene. Consequently, inhibiting telomerase function represents a significant strategy for suppressing cancer cell proliferation (9). Resveratrol has been extensively studied for its potential to inhibit telomerase activity and reduce the growth of cancer cells in different types of cancers.

Resveratrol has been shown to induce proliferation arrest and significantly reduce cell viability in the colon cancer cell lines HT-29 and WiDr. Telomerase activity (TLMA) is downregulated in a dose- and time-dependent manner, with inhibition reaching up to 94% in HT-29 cells and 93.1% in WiDr cells within 48 to 72 h. This inhibition disrupts telomere maintenance through hTERT destabilization, leading to apoptosis. Additionally, resveratrol may interfere with the cell cycle, potentially via the cyclin D1/Cdk4 pathways (84). In human cancer cells, resveratrol has been shown to inhibit important transcription factors responsible for activating the hTERT promoter, such as c-Myc and Sp1, thereby leading to reduced expression and activity of telomerase by lowering hTERT expression (85). Moreover, resveratrol attenuates downstream oncogenic signaling for regulating telomerase expression by inhibiting the PI3K/Akt signaling pathway and thereby reduces hTERT expression and telomerase activity in hepatocellular carcinoma cells by impairing the prosurvival signaling pathways (86).

These effects position resveratrol as a multi-target agent that limits the replicative potential of cancer cells and promotes apoptosis in colon cancer. However, the requirement for relatively high concentrations (≥40 μg/mL) indicates that improved formulation strategies are necessary for clinical application.

### 6.6 Cell angiogenesis

To maintain cell viability and growth, tumors, like normal organs, need a constant supply of oxygen, glucose, and other nutrients as well as a way to expel metabolic waste. These functions are fulfilled by the tumor-associated vasculature. For many tumor forms, angiogenesis—the creation of new blood vessels—is frequently triggered and clearly advantageous (87).

Besides inhibiting replicative immortality mediated by telomerase activity, resveratrol additionally affects angiogenesis-related signaling pathways; in other words, it inhibits immortality as well as angiogenesis. Through the reduction of the viability of endothelial cells and the hindrance of the biological processes necessary for the formation of new vessels, it exerts a strong anti-angiogenic property. The compound inhibits the adhesion and migration of endothelial cells, the remodeling of the extracellular matrix, and the formation of capillary tubes. It not only exerts an effect on the endothelial cell layer but also suppresses the pro-angiogenic signals of VEGF and the level of inflammatory mediators responsible for tumor angiogenesis. Remarkably, resveratrol exhibits increased anti-angiogenic effectiveness when paired with complementary natural substances, indicating its possible use in multi-target therapeutic approaches meant to reduce treatment-related inflammation while inhibiting tumor vascularization. By inhibiting VEGF expression and reducing the activity of pro-angiogenic factors such as HIF-1α, resveratrol demonstrates potent anti-angiogenic effects. This action restricts the blood supply necessary for tumor growth by decreasing endothelial cell proliferation and tube formation. As demonstrated by a review, resveratrol inhibits angiogenesis by blocking the PI3K/Akt/MAPK signaling pathways and downregulating VEGF and HIF-1α (88).

In a previous study, resveratrol concentrations ranging from 40 to 120 μM decreased the viability of human umbilical vein endothelial cells (HUVECs) by 35 to 100% in a dose-dependent manner and induced morphological changes at higher doses. Additionally, resveratrol inhibited matrix degradation by reducing MMP-2 activity by 35% and impaired endothelial cell adhesion and migration by 50 to 60%. It also disrupted vessel formation by inhibiting the formation of capillary-like tube structures by up to 90%. *Ex vivo* validation using rat aortic rings confirmed a 50% reduction in microvessel sprouting following treatment. Mechanistically, these anti-angiogenic effects are associated with the downregulation of MMP-2 and interference with endothelial functions, including adhesion, migration, and organization. These findings position resveratrol as a promising multi-target anti-angiogenic agent; however, the relatively high concentrations required underscore the need for improved delivery systems for clinical application (89).

A study investigated the synergistic anti-angiogenic effects of resveratrol and Ginkgolide on VEGF-induced signaling in colorectal cancer. The individual effects of these compounds in reducing endothelial cell proliferation, migration, and tube formation were demonstrated using human umbilical vein endothelial cells (HUVECs). When combined in a 1:3 ratio, their synergistic effect was confirmed by a combination index (CI < 1), indicating enhanced efficacy. In an HT-29 colon cancer mouse model, combined treatment with resveratrol at doses ranging from 80 to 960 mg/kg/day significantly inhibited tumor growth by over 50% and reduced microvessel density by approximately 65%. Furthermore, this combination therapy mitigated chemotherapy-induced inflammation by downregulating IL-6, COX-2, and TNF-*α*. These findings underscore the therapeutic potential of resveratrol as part of a multi-target natural strategy that not only inhibits angiogenesis but also alleviates treatment-related inflammation. The synergistic effects suggest that resveratrol, when used in conjunction with other natural agents, could serve as an effective and low-toxicity complement to standard cancer therapies (90).

### 6.7 Cell invasion and metastasis

Cancer cells migrate and become invasive. Programs for invasive growth allow cancer cells to spread throughout surrounding tissue as well as into blood and lymphatic arteries; these vessels thereafter act as conduits for distribution to both close and far-off anatomical locations, a process known as metastasis (9). Cancer metastasis is also enhanced by tumor angiogenesis due to the permissive vasculature. By focusing on several pathways that control cell adhesion, tumor-promoting inflammation, and the epithelial-to-mesenchymal transition (EMT), resveratrol inhibits the invasion and metastasis of cancer cells. In order to restore epithelial characteristics, decrease mesenchymal markers, and prevent matrix remodeling, it modulates important signaling networks, such as β1-integrin and AKT/GSK-3β/Snail pathways. Resveratrol’s multi-targeted potential in regulating cancer progression is highlighted by these mechanisms, which not only limit metastatic spread but also enhance apoptotic responses and potentiate the effects of other natural or pharmacologic agents. Mechanistically, it prevents epithelial-mesenchymal transition (EMT) by boosting E-cadherin levels and lowering mesenchymal marker expression. Furthermore, it inhibits the PI3K/Akt/NF-κB signaling pathway, which helps decrease the migration and invasion of cancer cells (88, 91). Resveratrol has demonstrated synergistic effects when combined with other natural agents, enhancing the regulation of invasion and anti-inflammatory responses in cancer models. One study showed that resveratrol (1–5 μM) inhibited colorectal cancer (CRC) cell invasion and metastasis by targeting β1-integrin signaling within a pro-inflammatory tumor microenvironment. Using a three-dimensional alginate model that included fibroblasts and T-lymphocytes, resveratrol reversed epithelial-to-mesenchymal transition (EMT) by increasing E-cadherin levels and decreasing mesenchymal markers such as vimentin and Slug. Silencing β1-integrin with antisense oligonucleotides (ASO) abolished the anti-metastatic effects of resveratrol and restored invasive behavior, confirming the critical role of this receptor. Furthermore, resveratrol reduced NF-κB activation and the expression of downstream proteins, including MMP-9, CXCR4, and FAK, which are known to facilitate metastasis. Resveratrol also enhanced the effects of NF-κB inhibition, suggesting a synergistic role in regulating invasion and inflammation. The study additionally reported an increase in caspase-3-mediated apoptosis, which was reversed when β1-integrin was blocked. These findings indicate that resveratrol’s anti-metastatic properties are closely linked to its modulation of β1-integrin signaling and related pathways. Since metastatic activity depends on β1-integrin-mediated pathways, this receptor represents a promising target for combination therapies in metastatic colorectal cancer (CRC) (92).

Supporting this mechanism, another study confirmed that resveratrol can inhibit metastasis through a different pathway, highlighting its multi-pathway potential in controlling cancer spread. A study conducted by Yuan demonstrated that resveratrol reduced metastasis in colon cancer by reversing epithelial-to-mesenchymal transition (EMT) via the AKT/GSK-3β/Snail pathway. In SW480 and SW620 colon cancer cells, resveratrol (15–240 μM) significantly decreased cell invasion and migration. *In vivo*, mice treated with resveratrol (150 mg/kg/day) exhibited fewer lung metastases and reduced expression of mesenchymal markers.

Mechanistically, resveratrol decreased N-cadherin and increased E-cadherin, vimentin, and Snail, indicating a reversal of epithelial-mesenchymal transition (EMT). It also suppressed phosphorylated AKT and GSK-3β, leading to the downregulation of Snail. Importantly, AKT1 knockdown mimicked the effects of resveratrol, whereas resveratrol lost its activity in AKT1-deficient cells, confirming the key role of AKT1 in mediating its effects (93). These findings support the use of resveratrol as a natural anti-metastatic agent targeting EMT, particularly in colorectal cancer. However, further clinical validation is needed to confirm its efficacy and optimize dosing strategies (94).

### 6.8 Metabolic reprogramming (Warburg effect)

The factors mentioned above, along with the metabolic reprogramming characteristic of cancer cells, are closely linked to the effect of resveratrol in inhibiting pathways such as PI3K/Akt and HIF-1α, thereby impacting the Warburg effect. The Warburg effect refers to the increased glucose consumption by cancer cells, which preferentially utilize glycolysis for energy production, unlike normal cells that favor oxidative phosphorylation. This effect is amplified by the heightened production of energy and biomaterials required for cancer cell growth. Resveratrol’s inhibition of glycolysis and activation of oxidative phosphorylation reduce the production of energy and biomaterials necessary for cancer cell proliferation, while protecting normal cells by restricting cancer cell growth through its cytostatic effect (87).

Resveratrol disrupts cancer cell metabolism by reversing the Warburg effect, revealing a key mechanism through which it limits tumor progression. A study by Saunier et al. demonstrated that resveratrol at a low concentration (10 μM for 48 h) induces a significant metabolic shift in colon cancer cells, reversing the Warburg effect and promoting oxidative phosphorylation (OXPHOS). Resveratrol significantly reduced lactate production, enhanced ATP generation by 20%, and suppressed pentose phosphate pathway (PPP) activity by 36%. These changes were associated with increased activity of the pyruvate dehydrogenase (PDH) complex, which directs pyruvate into mitochondrial respiration. Mechanistically, resveratrol upregulated the expression of PDP1, the phosphatase that activates PDH, and reduced phosphorylation at the PDHE1α(S232) site. This metabolic shift depended on mitochondrial calcium influx, as calcium chelators and mitochondrial Ca^2+^ uniporter blockers abolished the resveratrol effect. Additionally, the CaMKKβ–AMPK pathway played a crucial role, as inhibitors of AMPK or CaMKKβ reversed the oxidative switch. Importantly, this metabolic reprogramming occurred without significant cytotoxicity, indicating a cytostatic effect. These findings demonstrate that resveratrol not only inhibits cancer proliferation but also targets the metabolic flexibility of tumor cells, identifying the PDH complex as a novel metabolic vulnerability for anticancer strategies (95).

In addition to enhancing oxidative phosphorylation, this study demonstrates that resveratrol (1–100 μM) disrupts glucose metabolism in MCF-7 breast cancer cells by directly inhibiting 6-phosphofructo-1-kinase (PFK), a key regulatory enzyme in glycolysis.

Treatment for 24 h resulted in dose-dependent reductions in cell viability (approximately 40% at 100 μM), glucose uptake, and intracellular ATP levels, confirming the induction of energy stress.

Cancer cells. Mechanistically, resveratrol inhibited phosphofructokinase (PFK) activity in both cell lysates and purified enzyme assays, achieving approximately 50% inhibition at 15 μM. It reduced the formation of the active PFK tetramer, shifting the enzyme to an inactive dimeric state, even under high pH conditions or in the presence of typical positive regulators such as ADP and fructose 2,6-bisphosphate (F2,6BP). Interestingly, resveratrol also enhanced the inhibitory effects of ATP and citrate, two well-known metabolic suppressors. These findings suggest that resveratrol impairs glycolysis by altering the structural stability of PFK, contributing to energy depletion and cancer cell death. The ability to modulate a rate-limiting step in glycolysis highlights PFK as a novel metabolic target for resveratrol in cancer therapy (96).

Beyond direct enzyme inhibition, this study provides strong evidence that resveratrol inhibits glucose metabolism in cancer cells by targeting intracellular reactive oxygen species (ROS) and the hypoxia-inducible factor-1α (HIF-1α) signaling pathway. Resveratrol (50–150 μM) dose-dependently reduced glucose uptake (measured by ^18F-FDG PET) in Lewis lung carcinoma, HT-29 colon, and T47-D breast cancer cells. Importantly, the suppression of ^18F-FDG uptake was more significant than the reduction in cell number, indicating a direct metabolic effect. Resveratrol decreased intracellular ROS levels, which correlated strongly with decreased glucose uptake (r = 0.94). Using ROS scavengers or inducers confirmed that ROS modulation is both necessary and sufficient for the metabolic action of resveratrol. Mechanistically, resveratrol reduced HIF-1α accumulation and downregulated Glut-1, leading to suppressed glycolytic flux and lactate production. Additionally, resveratrol inhibited Akt phosphorylation, and inhibition of PI3K or mTOR mimicked its effects on glucose uptake. *In vivo* PET imaging confirmed reduced tumor FDG uptake in mice treated with resveratrol. These findings highlight resveratrol’s ability to reprogram cancer metabolism by modulating ROS levels and inhibiting the PI3K/Akt/mTOR–HIF-1α–Glut-1 axis, revealing a novel metabolic vulnerability in tumor cells. These metabolic changes may also contribute to the anti-proliferative effects of resveratrol observed in earlier studies, confirming the interconnected nature of cancer hallmarks. This not only suppresses glycolysis as a metabolic pathway but also indirectly impairs cancer cell proliferation by limiting the availability of energy and biosynthetic precursors required for rapid growth (97).

### 6.9 Immune modulation

One of the main characteristics of cancer is evasion of immune destruction, in which tumor cells suppress antitumor immunity through immune checkpoint activation, persistent inflammation, and the growth of immunosuppressive cell populations in the tumor microenvironment (2). By modifying both innate and adaptive immune responses, resveratrol opposes these mechanisms and aids in the restoration of efficient immune surveillance. Immunosuppressive immune cells are reprogrammed, pro-tumor inflammatory signaling is suppressed, and cytotoxic lymphocyte activity is increased. Resveratrol restricts tumor-mediated immune escape and promotes antitumor immune function through coordinated regulation of inflammatory pathways, macrophage polarization, T-cell differentiation, and natural killer cell activation, underscoring its potential use as an immunomodulatory adjuvant in cancer therapy.

Beyond disrupting cancer cell metabolism, Resveratrol exerts bidirectional immunomodulatory effects that may help restore immune surveillance against cancer. It targets both innate and adaptive immune responses through anti-inflammatory signaling and direct immune cell modulation. At the molecular level, resveratrol (5–100 μM) inhibits NF-κB and COX-2 pathways, reducing the production of inflammatory cytokines such as TNF-*α*, IL-6, and IL-1β. It also activates SIRT1, which deacetylates transcription factors like RelA/p65, contributing to reduced inflammation. In macrophages, resveratrol repolarizes tumor-associated macrophages (TAMs) from the immunosuppressive M2 to the pro-inflammatory M1 phenotype by suppressing IL-10 and STAT3 signaling. In T cells, low doses (1–25 μM) enhance CD8 + cytotoxicity while suppressing immunosuppressive Th17 and regulatory T cells (Tregs). In natural killer (NK) cells, very low concentrations (as low as 1.5 μM) increase NKG2D and perforin expression, enhancing tumor cell lysis, whereas higher concentrations (>50 μM) may induce apoptosis. Furthermore, resveratrol at 12.5–25 μM inhibits regulatory B cells (Bregs), reducing TGF-*β* levels and autoantibody production (98). Although dose-dependent and cell-type-specific effects are observed, these findings suggest that resveratrol can overcome immune evasion mechanisms used by tumors, making it a promising immunomodulatory agent. However, challenges related to bioavailability and precise dosing must be addressed before clinical translation.

While resveratrol’s bidirectional immunomodulatory effects on immune cells, such as NK cells, contribute to enhancing immune responses against cancer, research has shown that the combination of resveratrol and curcumin at bioavailable concentrations (5 μM each) has demonstrated significant synergistic effects in boosting immune responses and inhibiting cancer cell survival. While each compound alone showed modest cytotoxic effects in a limited number of cancer cell lines, their combination reduced cell viability across all tested lines, including prostate, colon, and breast cancers. On the immune level, the CUR + RES combo increased CD25 expression on both CD4 + and CD8 + T cells, indicating stronger immune activation. Although curcumin alone enhanced IFN-*γ* production, resveratrol appeared to balance this effect by increasing IL-10 production from regulatory T cells (Tregs), helping control excessive inflammation. Natural killer (NK) cell activity was also improved, as shown by upregulation of activating receptors (NKG2D, NKp30), downregulation of inhibitory receptors (NKG2A, KIRs, TIGIT), and enhanced degranulation (CD107a) against tumor cells. Additionally, monocyte/macrophage activation was supported by increased CD68 expression. Importantly, the combination was non-toxic to healthy PBMCs and reduced oxidative stress. These results suggest that resveratrol, especially in combination with other polyphenols, may act as an effective adjuvant to stimulate antitumor immunity while preserving immune balance (99).

Although the combination of resveratrol and curcumin has shown positive immune effects in cellular models, a recent study demonstrates that resveratrol can enhance the anti-cancer function of natural killer (NK) cells, both *in vitro* and *in vivo*. At a concentration of 20 μM, resveratrol significantly enhanced IFN-*γ* secretion when combined with IL-2 (5 ng/mL) and improved NK cell cytotoxicity by over 21% at a 1:1 effector-to-target (E: T) ratio. Mechanistically, it upregulated activating receptors, such as NKG2D and NKp30, which promoted NK cell degranulation, as evidenced by increased CD107a expression. In a melanoma mouse model, resveratrol administered at a dosage of 0.5 mg/kg resulted in a reduction of tumor volume and lung metastasis. However, depletion of NK cells resulted in the loss of the anti-tumor effects of resveratrol, confirming the central role of these cells in its mechanism. Similar immune activation was observed in human samples, where 20 μM of resveratrol increased NK cell cytotoxicity by approximately 25%. These findings suggest that resveratrol enhances innate immune surveillance and may serve as a supportive agent in cancer immunotherapy. However, its rapid metabolism underscores the necessity for optimized delivery systems to maintain effective concentrations *in vivo* (100).

### 6.10 Tumor-induced inflammation

Tumor-induced inflammation, a well-established hallmark of cancer, promotes tumor initiation, progression, angiogenesis, immune evasion, and metastasis through persistent activation of pro-inflammatory signaling pathways. Inflammatory mediators produced by cancer cells and stromal components within the tumor microenvironment activate transcription factors such as NF-κB and STAT3, which enhance the invasion and survival of malignant cells. Consequently, targeting inflammation-driven signaling pathways has become crucial for both cancer treatment and prevention. Resveratrol exhibits potent anti-inflammatory properties by modulating key inflammatory pathways and immune cell responses, making it a promising agent for inhibiting the progression of inflammation-associated tumors (101).

A recent study shows that resveratrol effectively reduces inflammation in LPS-stimulated macrophages by targeting key pro-inflammatory pathways. At concentrations between 1 and 20 μM, resveratrol dose-dependently inhibited the production of nitric oxide (NO) and interleukin-6 (IL-6), two key inflammatory mediators. The highest effect was observed at 20 μM, without causing cell toxicity. Mechanistically, resveratrol blocked the translocation of High-mobility group box 1 (HMGB1) from the nucleus to the cytoplasm, a crucial early step in the inflammatory response. It also suppressed the nuclear transcription factor kappa-B(NF-κB) pathway by preventing p65 translocation to the nucleus and reducing phosphorylation of IκBα. In parallel, resveratrol inhibited the JAK/STAT signaling pathway by decreasing the phosphorylation of STAT1 and STAT3. These effects were confirmed by pharmacological inhibitors, PDTC for NF-κB and AG490 for JAK2, which showed similar results. Together, these findings demonstrate that resveratrol has a strong anti-inflammatory action by simultaneously blocking two central pathways of inflammation. This supports its potential as a therapeutic agent for inflammation-driven cancers and other inflammatory diseases (102).

Additionally, resveratrol decreased STAT3 phosphorylation and transcriptional activity in several tumor models, which resulted in a reduction in the expression of STAT3-dependent genes related to immune suppression, angiogenesis, and survival. In the tumor microenvironment, this effect leads to decreased tumor growth and increased immune responsiveness (103). Resveratrol influences the inflammatory tumor microenvironment by modulating macrophage polarization, in addition to directly inhibiting signaling pathways. Research indicates that resveratrol reduces tumor-promoting inflammation by enhancing pro-inflammatory, tumor-suppressive M1 macrophage traits while suppressing M2-like tumor-associated macrophage phenotypes (104).

### 6.11 Genomic mutations and instability

One of the primary hallmarks of cancer is genomic instability, which promotes tumor development and progression by accumulating DNA damage and impairing repair mechanisms. Resveratrol has been shown to preserve genome integrity by reducing DNA damage and enhancing DNA repair processes, building on its well-known anti-inflammatory properties (87). A previous study demonstrated that resveratrol helps protect genome integrity and may prevent cancer by decreasing DNA damage and supporting DNA repair pathways. At low concentrations (1.25–2.5 μM), resveratrol reduced the formation of DNA double-strand breaks (DSBs) in mouse embryonic fibroblasts, as evidenced by a 50% reduction in *γ*-H2AX foci after 24 h. Mechanistically, it transiently stabilized histone H2AX, a critical factor in DSB repair, and alleviated replication stress, resulting in fewer cells entering uncontrolled proliferation. In a cancer-prone mouse model (Msh2-deficient), dietary supplementation with melinjo-derived resveratrol (0.03%) decreased intestinal tumor incidence and extended lifespan by 30%. Related polyphenols, such as chlorogenic acid and gnetin C, exhibited similar DSB-reducing effects, reinforcing the concept that natural dietary compounds can maintain genome stability. Importantly, these protective effects were observed at physiologically achievable doses, unlike higher concentrations (≥25 μM), which may induce apoptosis. These findings support the role of resveratrol as a genomic stabilizer, particularly in cancers associated with microsatellite instability or defective DNA repair (105).

**Figure 4:**
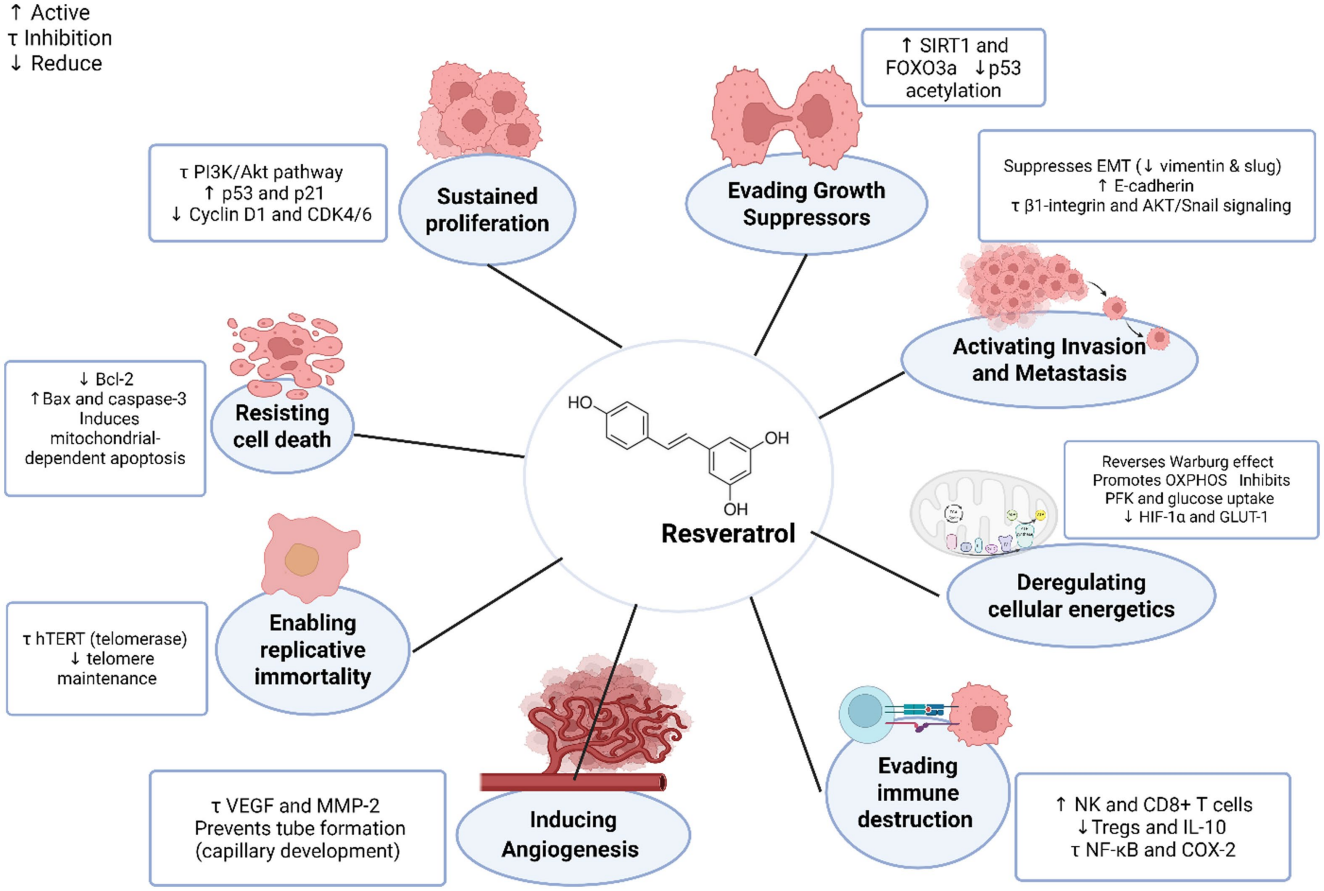
Mechanisms of resveratrol as an anticancer agent. RES, resveratrol; PI3K, phosphatidylinositol-3-kinase; AKT, protein kinase B; MAPK, mitogen-activated protein kinase; ROS, reactive oxygen species; MMP, matrix metalloproteinase; VEGF, vascular endothelial growth factor; EMT, epithelial–mesenchymal transition; NF-κB, nuclear factor kappa-B.

In addition to safeguarding genome integrity, a significant study reveals that trans-resveratrol (RSV), a natural polyphenol found in red wine, stimulates the transcription of the human MCM4 gene, which is essential for DNA replication and genome stability. Researchers employed luciferase reporter assays and found that RSV at a concentration of 20 μM significantly enhanced MCM4 promoter activity in HeLa S3 and HL-60 cells. This activation depends on the presence of GGAA (c-ETS) motifs and a GC-box, which are critical regulatory elements. Mutations in these motifs abolished the response, confirming their importance. RT-qPCR analysis showed that MCM4 mRNA levels increased within 2–4 h of RSV treatment, while western blotting revealed a peak in MCM4 protein levels at 24 h. Electrophoretic mobility shift assays identified the transcription factors Sp1 and PU.1 as key regulators binding to the promoter region. RSV also shifted the Sp1/PU.1 ratio in favor of Sp1, correlating with enhanced gene transcription. These effects were observed in both p53-positive and p53-deficient cells, indicating a p53-independent mechanism. The findings suggest that low, non-toxic doses of RSV (20 μM) can modulate MCM4 expression and may contribute to maintaining genome stability, providing insights into the chemopreventive potential of resveratrol (106) (Figure 4).

## 7 Resveratrol derivatives and structure–activity relationship

To enhance the pharmacological effects and pharmacokinetics of resveratrol, increasing focus has been placed on creating its derivatives. To circumvent the limitations of the parent substance, synthetic analogues of resveratrol have been developed, and it has been demonstrated that structural modifications influence bioavailability, stability, and anticancer activity by altering the core framework and substituent patterns. Numerous studies have shown that these compounds exhibit improved pharmacokinetic properties and enhanced cytotoxic effects in cancer models compared to resveratrol alone (107). Trimethoxy-resveratrol (TMS), a methoxylated derivative of resveratrol, has demonstrated greater efficacy against cancer than resveratrol. The presence of methoxy groups increases lipophilicity and metabolic stability, which encourages increased cellular uptake and stronger cytotoxic effects in cancer cells. Multiple findings report that TMS induces greater inhibition of cancer cell growth, cell cycle arrest, reduced metastasis, decreased angiogenesis, and increased apoptosis, indicating a more potent anticancer profile than resveratrol (108, 109). Polydatin (piceid), the glycosylated form of resveratrol, exhibits improved cellular transport and chemical stability. Glycosylation enhances metabolic stability and aqueous solubility, potentially leading to increased bioavailability. Several studies have shown that polydatin inhibits cancer cell growth and induces apoptosis, effects that are partially mediated by the modulation of oxidative stress and inflammatory signaling pathways (110, 111). Additionally, oxyresveratrol, a hydroxylated derivative of resveratrol with additional phenolic hydroxyl groups, demonstrates increased antioxidant capacity. The presence of extra hydroxyl groups has been associated with apoptotic and antiproliferative effects in cancer cells, as well as enhanced free-radical scavenging activity. These findings support the notion that hydroxyl substitution enhances the anticancer potential of resveratrol derivatives (112, 113).

Combinational approaches involving resveratrol have demonstrated promising synergistic anticancer effects, alongside its structural analogs. These approaches involve combining resveratrol or resveratrol-inspired compounds with conventional chemotherapeutic drugs or targeted therapies to produce a synergistic anticancer impact via various cellular signaling pathways, as evident in recent literature reviews on this topic (114).

For example, resveratrol enhanced the efficacy of docetaxel and platinum-based chemotherapeutics (cisplatin, carboplatin) by inhibition of cancer cell proliferation and ROS generation in A549 lung adenocarcinoma cells (115). Additionally, resveratrol has been demonstrated to increase cisplatin-induced apoptosis by regulating autophagy in lung cancer cells, further emphasizing its ability to make tumors more responsive to conventional chemotherapy treatments (116). When combined with 5-fluorouracil, it increased apoptosis and growth inhibition in colorectal and glioblastoma models (117). Proanthocyanidins, natural phytochemicals found in grape seeds, work synergistically with resveratrol to induce apoptosis and epigenetic modifications (118). Furthermore, the co-delivery of paclitaxel and resveratrol via nanocarriers exhibited enhanced cytotoxicity and efficacy against drug-resistant tumors, highlighting the therapeutic potential of co-delivery and combinational strategies (119).

## 8 Formulation of resveratrol

Resveratrol exhibits very low oral bioavailability—less than 1%—due to rapid and extensive metabolism in the intestine and liver, resulting in the formation of sulfate and glucuronide metabolites. However, its oral absorption is relatively high, at approximately 75%. Consequently, only a small fraction of unmetabolized resveratrol reaches the bloodstream following oral administration (120, 121).

Other significant obstacles include the low aqueous solubility of resveratrol (less than 1 mg/mL) and its susceptibility to oxidation and extreme photosensitivity (122). To enhance the bioavailability, half-life, solubility, and stability of resveratrol, numerous formulations have been explored. These include microparticles, complexation with cyclodextrin, vesicular systems such as liposomes, and various nanocarrier systems, including solid lipid nanoparticles (SLNs) (Table 2).

**Table 2:** Impact of various resveratrol delivery methods on its bioavailability.

Reference	Formulation type	Administration route	Bioavailability outcome	Quantitative bioavailability data
Gartziandia et al. (123)	Pectin/alginate gastro-resistant microparticles	Oral (*in vitro* + *in vitro* cell assays)	Gastro-resistance confirmed; bioactivity preserved	No *in vivo* BA data
Penalva et al. (124)	Zein nanoparticles	Oral (mouse model)	Improved systemic exposure and anti-inflammatory effect	↑ AUC and plasma levels (fold increase reported)
Spogli et al. (125)	Magnesium dihydroxide-supported microparticles (solid dispersion)	Oral (rabbit model)	Significantly improved oral bioavailability	~3.3-fold increase in AUC
Briskey and Rao (126)	LipiSperse™ lipid dispersion (microparticulate system)	Oral (human study)	Enhanced absorption in humans	~2–3-fold increase in plasma exposure
Wang et al. (127)	Cyclodextrin inclusion complex	Pulmonary	Improved stability and lung delivery	No systemic BA fold reported
Kong et al. (128)	HP-β-cyclodextrin inclusion complex	Pulmonary	Improved lung disposition and metabolic stability	No oral BA comparison
Jhaveri et al. (129)	Transferrin-targeted liposomes	Intravenous	Enhanced brain delivery and tumor targeting	No oral BA data
Vijayakumar et al. (130)	Vitamin E TPGS-coated liposomes	Intravenous	Improved circulation time and tissue distribution	BA fold not reported
Wang et al. (131)	Solid lipid nanoparticles	*In vitro* only	Enhanced anticancer activity	No pharmacokinetics
Pandita et al. (132)	Solid lipid nanoparticles	Oral (rat model)	Markedly enhanced oral bioavailability	~8-fold increase in BA
Brotons-Canto et al. (133)	Zein nanoparticles	Oral (human study)	Improved plasma exposure and half-life	↑ AUC and residence time

### 8.1 Microparticle-based formulation

Micro- and nano-delivery systems have been extensively studied as strategies to overcome the limitations of resveratrol by preventing its premature degradation, controlling its release, and potentially enhancing its absorption. Microencapsulation, including gastro-resistant coatings, can protect resveratrol as it passes through the gastrointestinal tract. Targeted release in higher pH environments, such as the distal intestine, may improve its local bioaccessibility and stability (123).

In a study conducted by Oihane Gartziandia, resveratrol was encapsulated in Eudragit FS-30D-coated pectin-alginate microparticles. This gastro-resistant formulation for oral administration improves bioavailability and offers protection. The capsule was evaluated for dissolution and drug release. As an example of their potential to enhance intestinal delivery, gastro-resistant pectin/alginate microparticles coated with Eudragit FS-30D protected resveratrol at acidic pH (with less than 10% release) and facilitated its release at intestinal pH (approximately 70% over 24 h) without compromising its biological activity *in vitro*. However, this study did not demonstrate improved systemic bioavailability of the encapsulated resveratrol, as it lacked *in vivo* pharmacokinetic data (123). Although microencapsulation enhances stability, enables controlled release, and sometimes improves *in vitro* bioaccessibility, there is still limited evidence in the literature that microparticle formulations increase oral bioavailability *in vivo*. Reviews of resveratrol delivery systems indicate that microparticulate carriers improve chemical stability and reduce degradation; however, no clinical or animal pharmacokinetic studies have demonstrated increased systemic exposure following microparticle administration. On the other hand, several nanocarrier-based strategies, such as protein- or polymer-based nanoparticles, have demonstrated enhanced bioavailability *in vivo*. For example, in mice, resveratrol loaded into zein nanoparticles exhibited increased plasma levels and relative oral bioavailability—approximately 19 times higher than that of a control solution—indicating that nanoscale formulation strategies can significantly improve systemic exposure (124). Overall, rigorous *in vivo* studies measuring bioavailability improvement—such as plasma AUC or Cmax comparisons—remain necessary to support claims of increased systemic availability, even though microencapsulation techniques like gastro-resistant microparticles are promising for protecting resveratrol and improving its release characteristics. Future research should compare microparticulate and nanoparticulate delivery systems directly in pharmacokinetic models in an effort to close this gap.

### 8.2 Solid dispersion formulation

In 2018, Roberto Spogli conducted a study aimed at enhancing the solubility and bioavailability of resveratrol using a solid dispersion method with magnesium dihydroxide (Resv@MDH). Dissolution tests comparing Resv@MDH to free resveratrol revealed that Resv@MDH exhibited a dissolution rate five times faster and a maximum solubility three times greater than that of free resveratrol. Additional dissolution tests against pure micronized resveratrol with similarly sized particles showed that while reducing particle size influenced dissolution kinetics, it did not affect maximum solubility. For absorption assays, *in vivo* tests were performed on rabbits to measure plasma concentrations of resveratrol following administration of 50 mg/kg of Resv@MDH and pure resveratrol. The results indicated that Resv@MDH achieved a higher peak plasma concentration (Cmax: 101.3 ng/mL) compared to pure resveratrol (76.3 ng/mL), with faster absorption occurring within 15 to 30 min post-administration. Furthermore, plasma levels of resveratrol from Resv@MDH remained significantly elevated relative to the free form for up to 90 min. Area under the curve (AUC) analysis demonstrated a 3.3-fold increase in systemic exposure for Resv@MDH, underscoring its potential to enhance the oral bioavailability of resveratrol (125). Similarly, in a single-dose, randomized pharmacokinetic study conducted on healthy volunteers, a resveratrol formulation containing LipiSperse® exhibited approximately twice the plasma AUC and three times the Cmax of trans-resveratrol conjugates compared to a standard resveratrol product, suggesting improved oral bioavailability (126).

### 8.3 Cyclodextrin-based formulation for stability and pharmacokinetic enhancement

In 2020, Xuechun Wang conducted research that aimed at enhancing the aqueous solubility and stability of resveratrol by forming a complex with sulfobutylether-*β*-cyclodextrin (SBECD) in a 1:1 ratio (SBECD: RES). The study focused on formulation considerations and the impact on non-small cell lung cancer (NSCLC) cell lines. The stability of the inclusion complex in phosphate-buffered saline (PBS) at pH 7.4 showed that pure resveratrol was less stable than the CD-RES complex; after 12 h, over 50% of the pure resveratrol had degraded, while the CD-RES complex retained more than 90% of the drug after 24 h and over 80% after 192 h. While resveratrol followed first-order degradation kinetics, the CD-RES complex exhibited no significant degradation for up to 72 h, indicating that cyclodextrin complexation enhances the stability of resveratrol. Further investigations into the stability of resveratrol and CD-RES in biological media revealed that free resveratrol rapidly degraded, with less than 2% remaining after 96 h at 37 °C, whereas the CD-RES complex preserved approximately 26.2 and 32.9% of resveratrol in rat and human plasma, respectively. Additionally, CD-RES exhibited detectable levels of resveratrol that were 2 to 3 times higher after 24 h compared to the free form. Both forms followed first-order degradation kinetics; however, the half-life of resveratrol was significantly prolonged due to cyclodextrin complexation—from 14.1 h to 48.3 h in rat plasma (approximately a 3.4-fold increase) and from 15.0 h to 60.1 h in human plasma (approximately a 4-fold increase). These findings highlight the protective effect of cyclodextrin on resveratrol in biological environments. To further assess whether the complex affected the biological activity, the MTT assay was employed to assess the cytotoxic activity of resveratrol (RES) and its cyclodextrin complex (CD-RES) against five non-small cell lung cancer (NSCLC) cell lines *in vitro*. Both formulations demonstrated comparable cytotoxic effects across a broad concentration range (0.39–100 μM). IC₅₀ values indicated that CD-RES exhibited reduced cytotoxicity in certain cell lines compared to free resveratrol. Notably, in H4006 and H358 cells, the IC₅₀ values for CD-RES were significantly higher (684.8 ± 183.3 μM vs. 133.4 ± 17.1 μM in H4006 and 131.3 ± 39.6 μM vs. 50.0 ± 19.1 μM in H358), suggesting a potential decrease in anticancer efficacy upon complexation (127). Compared to oral delivery, pulmonary administration of a resveratrol–hydroxypropyl-*β*-cyclodextrin inclusion complex in rats demonstrated enhanced absorption and higher bioavailability (~92.95%), characterized by rapid uptake and increased lung exposure (128).

### 8.4 Liposomal delivery

Nowadays, liposomal delivery is one of the most recognized and advanced techniques. Significant quantitative benefits of the targeted formulation were demonstrated in *in vivo* xenograft studies. The average tumor volumes on day 18 were 1018.8 ± 87.5 mm^3^ with PBS, 728.2 ± 117.9 mm^3^ with free resveratrol, 506.9 ± 69.6 mm^3^ with RES-L, and 349.3 ± 38.1 mm^3^ with Transferrin resveratrol loaded liposome/nanoparticle (Tf-RES-L). This corresponds to an approximate 52% reduction compared to the free drug. Additionally, median survival analysis showed that Tf-RES-L extended survival to 28 days, compared to 22 days for free resveratrol. Furthermore, while only about 20% of the free resveratrol or RES-L groups had not reached the predefined tumor cutoff by day 25, approximately 75% of the Tf-RES-L group remained below this threshold. These quantitative results demonstrate that transferrin-targeted liposomes enhance drug delivery and therapeutic efficacy (129). Similarly, TPGS-coated liposomal formulations of resveratrol significantly enhanced its systemic exposure *in vivo*. In a rat pharmacokinetic study, TPGS-coated liposomes (RSV-TPGS-Lipo 2) exhibited approximately a 30-fold increase in AUC and a longer half-life compared to free resveratrol. These results suggest that the bioavailability of resveratrol was substantially improved using this delivery method (130).

### 8.5 Solid lipid nanoparticles

The objective of Wang (131) study on solid lipid nanoparticles (SLNs) was to develop resveratrol-loaded SLNs (Res-SLNs) for the treatment of breast cancer. The study also evaluated the effects of SLNs on MDA-MB-231 human breast cancer cells. The sulforhodamine B (SRB) assay was employed to assess *in vitro* cytotoxicity, and the half-maximal inhibitory concentration (IC_50_) was calculated. Results indicated that Res-SLNs exhibited greater cytotoxicity against the cells compared to free resveratrol. Additionally, Res-SLNs demonstrated a superior ability to induce cell death compared to free resveratrol, resulting in a higher percentage of G0/G1 cell cycle arrest (25.5 ± 1.38%) compared to free resveratrol (19.2 ± 0.68%) under identical conditions. Furthermore, Res-SLNs inhibited the invasion and migration of MDA-MB-231 cells more effectively than free resveratrol (131). *In vivo*, solid lipid nanoparticle (SLN) systems have been shown to improve oral bioavailability. Stearic acid-based resveratrol SLNs coated with poloxamer 188, when administered *in vivo* to Wistar rats, resulted in an approximately 8.03-fold increase in oral bioavailability compared to a free resveratrol suspension. This improvement is attributed to enhanced solubility, protection from degradation, and sustained release (132). Other nanoparticle systems beyond solid lipid nanoparticles (SLNs) have also demonstrated enhanced systemic exposure. Human pharmacokinetic studies have shown that resveratrol nanoparticle dispersions prepared with zein result in a substantial increase in both resveratrol exposure and its half-life compared to resveratrol alone (133).

## 9 Clinical applications of resveratrol in human: study designs, limitations, dose rationale, sample size interpretation

In a clinical study involving 20 colorectal cancer patients, oral resveratrol administered at doses of 0.5 or 1.0 g /day for eight days resulted in significant tissue accumulation, particularly in the right colon. Although plasma levels of the parent compound were low, metabolites such as glucuronides and sulfates were detected in both plasma and tissue, confirming rapid metabolism while maintaining a sustained local presence. High-performance liquid chromatography and mass spectrometry revealed tissue concentrations of up to 674 nmol/g for resveratrol and 86 nmol/g for its metabolites. Tumor tissue exhibited a 5% reduction in Ki-67 staining, indicating decreased cell proliferation. Although modest, this antiproliferative effect corroborates previous preclinical findings. The study demonstrates that oral dosing achieves concentrations exceeding the *in vitro* IC₅₀ values for colon cancer cells, suggesting that resveratrol may function as a safe and biologically active chemopreventive agent. Variations in regional tissue exposure also highlight the potential for personalized delivery strategies. While the study was limited by its small sample size and short duration, it provides a compelling rationale for further trials investigating long-term effects and optimized formulations (134).

Building on findings regarding the bioavailability and antiproliferative effects of resveratrol in colorectal cancer patients, a Phase I pilot clinical trial was conducted involving eight patients with colon cancer. This trial evaluated the effects of plant-derived resveratrol (20 or 80 mg/day) and resveratrol-rich grape powder (80 or 120 g/day) administered over 14 days before surgery. While isolated resveratrol produced minimal changes, grape powder significantly inhibited Wnt pathway gene expression—including cyclin D1 and axin II—in normal colonic mucosa (*p* < 0.001), although no such effect was observed in tumor tissue. Interestingly, some oncogenes, such as MYC and cyclin D1, were upregulated in tumor samples. Furthermore, grape powder reduced the expression of stem cell markers (CD133, LGR5) in normal tissue, suggesting potential chemopreventive effects. These findings underscore the importance of polyphenol synergy in whole-food formulations like grape powder and support their role in early-stage cancer prevention rather than in treating established cancer. However, the small sample size limits the generalizability of these results and necessitates further investigation (135). While the use of grape powder in colon cancer patients has demonstrated potential benefits for early-stage prevention, a randomized open-label trial assessed the effects of liposomal resveratrol (400 mg/day) in 40 patients with head and neck cancer (HNC) receiving home enteral nutrition (HEN) over 12 weeks. The treatment group showed a significant increase in glutathione peroxidase (GPx) activity (*p* = 0.02) and an improved phase angle (PhA) (*p* = 0.004), indicating enhanced antioxidant defense and cellular integrity. Although total antioxidant capacity (TAC) and superoxide dismutase (SOD) levels increased in both groups—likely due to HEN—the lipid peroxidation marker malondialdehyde (MDA) rose more sharply in the resveratrol group. This suggests a dual antioxidant and pro-oxidant effect, particularly at higher doses. Fat mass remained stable in the resveratrol group but increased in the control group, suggesting possible metabolic benefits. Overall, this study supports the safe use of liposomal resveratrol in supportive cancer care while underscoring the need to optimize dosing to prevent oxidative imbalance (136).

However, liposomal resveratrol has shown potential in enhancing antioxidant status in cancer patients. Two clinical trials investigated the effects of SRT501, a micronized resveratrol formulation developed to improve bioavailability. In a Phase I study involving nine colorectal cancer patients with liver metastases, oral administration of SRT501 (5 g/day for 14 days) achieved plasma levels 3.6 times higher (Cmax = 1,942 ng/mL) than those of standard resveratrol and penetrated liver tumors at concentrations up to 2,287 ng/g. Additionally, a 39% increase in cleaved caspase-3 (*p* = 0.038) confirmed apoptotic activity in the tumor tissue (137).

In another Phase II trial involving 24 patients with multiple myeloma, SRT501 combined with bortezomib demonstrated limited efficacy, with an overall response rate ranging from 8 to 22%. The study was associated with renal toxicity in five patients, which led to early termination. These results highlight both the therapeutic potential and safety concerns related to high-dose resveratrol in advanced cancers, particularly among vulnerable patient populations. Future studies should focus on developing safer formulations and combination protocols to improve tolerability (138).

In addition to its effects in cancer patients, resveratrol has demonstrated potential benefits for individuals at higher risk of developing cancer. For example, supplementation with 50 mg twice daily for 12 weeks reduced DNA methylation of the tumor suppressor gene Ras association domain-containing protein 1 (RASSF1A) in the breast tissue of women at increased risk of breast cancer (139). Furthermore, resveratrol supplementation at 1 g/day for 12 weeks increased concentrations of sex hormone-binding globulin (SHBG), which has been associated with a reduced risk of breast cancer (140), and positively influenced estrogen metabolism. These findings suggest that resveratrol may reduce breast cancer risk factors in obese and overweight postmenopausal women (141).

Another clinical study focused on resveratrol’s effects on potential biomarkers associated with cancer risk. Elevated circulating levels of insulin-like growth factor 1 (IGF-1) and IGF-binding protein 3 (IGFBP-3) have been linked to an increased risk of several common cancers (142). Brown et al. demonstrated that administering resveratrol at 2.5 g/day for 29 days reduced the circulating levels of IGF-1 and IGFBP-3 in healthy volunteers (59). This suggests that resveratrol’s ability to lower circulating IGF-1 and IGFBP-3 in humans may represent a potential anti-carcinogenic mechanism (Table 3).

**Table 3:** Resveratrol in anticancer clinical studies.

Study (Population)	Study design	Dose and duration	Dose rationale	Primary endpoint(s)	Limitations	Sample size interpretation
Patel et al. (134) (CRC patients pre-surgery)	Interventional PK/PD tissue study (*n* = 20)	0.5 g or 1.0 g daily × 8 days	Dose choice based on prior phase I repeat-dose study in healthy volunteers showing tolerability at 0.5–1.0 g	Resveratrol and metabolite levels in colorectal tissue; apoptosis markers	Non-randomized, no placebo control Short intervention duration (8 days) High inter-individual variability in tissue concentrations Limited ability to confirm long-term clinical benefit	Small exploratory sample (*n* = 20) appropriate for pharmacokinetic and tissue-distribution objectives, but underpowered for definitive clinical efficacy conclusions.
Nguyen et al. (136) (colon cancer pts) (135)	Phase I open-label pilot (*n* = 8)	20 or 80 mg resveratrol OR grape powder for 14 days	Time window matched diagnosis-to-surgery interval	Tumor cell proliferation (Ki-67)	Very small sample; difficulty recruiting participants; no control arm	Sufficient only for feasibility and hypothesis generation; no reliable efficacy inference
Ławiński et al. (136) (head and neck cancer patients on HEN)	Randomized, interventional, open-label, single-center clinical study	40 patients (20 RES, 20 control)	400 mg chosen based on prior clinical trials, safety considerations (<1 g/day),	↑ GPx and Phase Angle only in RES group; ↑ TAC and SOD in both groups; ↑ MDA more pronounced in RES group (suggesting dual antioxidant/pro-oxidant activity)	Small final sample size; high dropout; open-label design (risk of bias); single-center; short intervention duration (12 weeks); baseline imbalance in TAC	Final sample (*n* = 40) limits statistical power and generalizability; adequate for exploratory mechanistic signals, not definitive clinical efficacy
Howells et al. (137) (CRC with liver metastases)	Phase I randomized, double-blind pilot (*n* = 7 completers)	Micronized resveratrol (SRT501) 5 g/day for ~10–21 days	Micronization to improve bioavailability	Hepatic tissue resveratrol levels; apoptosis markers	Small sample; variable dosing duration; perioperative complications	Adequate to show detectability in liver tissue and preliminary PD signals; not safety or efficacy
Popat, et al. (138) (multiple myeloma patients)	Phase 2 clinical trial using an adaptive strategy: all patients started on SRT501 monotherapy, with bortezomib added upon progressive disease (PD) after initial cycles. (*n* = 13)	SRT501 5.0 g (micronized resveratrol) daily after breakfast for 20 days of a 21-day cycle, up to 12 cycles. Bortezomib 1.3 mg/m^2^ on days 1,4,8,11 added for PD.	Resveratrol has poor solubility/bioavailability, so micronized SRT501 was developed. The Phase 2 dose (5.0 g) had been “safely assessed” in prior SRT501 studies (mainly non-cancer cohorts).	Limited efficacy (8–22% response); renal toxicity in 5 pts. → early termination	Early termination driven by unexpected renal toxicity: 5 serious AEs of renal failure prompted stopping recruitment. High attrition/withdrawal: many discontinued due to AEs and decisions, reducing evaluable numbers and precision. Crossover/adaptive design complicates interpretation.	Although designed to estimate response (planned 30 evaluable), the trial enrolled 24 with only 13 evaluable, and was stopped early for safety, meaning the study is underpowered and imprecise for efficacy and primarily supports conclusions about unacceptable safety in this population.
Zhu et al. (139) (women at ↑ breast cancer risk)	Randomized, double-blind, placebo-controlled (*n* = 39)	5 mg or 50 mg BID × 12 weeks	Common supplemental doses; BID due to rapid metabolism	DNA methylation, PGE2 levels	Modest sample; surrogate endpoints	Adequate for directional biomarker changes; underpowered for risk reduction outcomes
Chow et al. (141) (overweight postmenopausal women)	Open-label, single-arm trial (*n* = 34 completers)	1 g daily × 12 weeks	Focus on hormonal modulation in high-risk group	Estradiol, estrogen metabolites, inflammatory markers	No control group; liver enzyme elevation in one participant	Sufficient for within-subject biomarker change, not causal inference or safety generalization
Brown et al. (59) (healthy volunteers)	Repeat-dose dose-escalation study (*n* = 40; 10/group)	0.5–5.0 g daily × 29 days	Evaluate safety, PK, and IGF-axis modulation across doses	Safety, pharmacokinetics, IGF-1 / IGFBP-3 changes	Healthy population; GI intolerance at higher doses	Sufficient for dose-related PK, tolerability, and biomarker trends; not cancer prevention efficacy

Although *in vitro* studies have demonstrated that resveratrol exerts anticancer effects through several mechanisms—including the induction of apoptosis, inhibition of cell proliferation, and modulation of signaling pathways—translating these findings to humans remains uncertain. Clinical studies have shown tissue accumulation and modulation of proliferation; however, these effects are modest and dose-dependent. Additionally, factors such as metabolism, tissue distribution, and formulation can influence its efficacy. Therefore, further clinical research is necessary to clarify the potential of resveratrol for cancer prevention and as a therapeutic agent in humans.

## 10 Conclusion

Resveratrol—a naturally occurring polyphenolic phytoalexin—has garnered extensive scientific research and numerous patents due to its numerous health-promoting benefits. For over two millennia, plants containing resveratrol have been effectively used in traditional medicine. Its potent anti-inflammatory and antioxidant properties make it a valuable agent for treating various ailments. Certain plants, fruits, and derivatives, such as red wine, are rich sources of resveratrol. Consequently, it can be administered through nutraceutical tablets or by consuming these natural components. Resveratrol acts as a multi-target anticancer agent, as demonstrated by preclinical, mechanistic, and clinical studies. It disrupts many hallmark features of cancer, including immune evasion, metabolic reprogramming, angiogenesis, invasion, resistance to apoptosis, sustained proliferation, and genomic instability. By modulating signaling pathways such as PI3K/Akt, NF-κB, SIRT1, and JNK, as well as through epigenetic regulation, mitochondrial targeting, and anti-inflammatory effects, resveratrol exhibits both preventive and therapeutic potential. Despite strong preclinical evidence supporting resveratrol’s anticancer properties, challenges remain for its successful clinical application. Greater focus is needed on resveratrol analogs and derivatives, such as methoxylated or glycosylated resveratrols, which have demonstrated improved stability, bioavailability, and anticancer efficacy compared to the original compound. To overcome challenges such as low oral bioavailability, rapid metabolism, and high interindividual variability, appropriate drug formulations and pharmacokinetic studies are essential. Standardizing dosages, formulations, and modes of administration across ongoing studies is crucial to better evaluate resveratrol’s anticancer potential.

## Glossary

Akt: Protein kinase B
AMPK: AMP-Activated Protein Kinase
APE 1: Apurinic/Apyrimidinic Endonuclease 1
CaMKKβ: Calcium/Calmodulin-Dependent Protein Kinase Kinase Beta
CD95: Cluster of differentiation 95
Cdk4: Cyclin-Dependent Kinase 4
c-Myc: cellular Myc proto-oncogene
COX-2: Cyclooxygenase 2
CRC: Colorectal cancer
CXCR4: C-X-C Motif Chemokine Receptor 4
Cyclin D1: G1 phase regulatory cyclin
DMH: 1,2-Dimethylhydrazine
ECM: Extracellular Matrix
EGFR: Epidermal Growth Factor Receptor
EMT: Epithelial–mesenchymal transition
eNOS: Endothelial nitric oxide synthase
FAS: Cell Surface Death Receptor
FDG: Fluorodeoxyglucose
FOXO: Forkhead box O
GLUT1: Glucose Transporter 1
GPx: Glutathione peroxidase
HIF-1α: Hypoxia-inducible factor-1α
hTERT: Human telomerase reverse transcriptase
IC₅₀: Half-maximal inhibitory concentration
IGF-1: Insulin-like growth factor-1
IGFBP-3: IGF-binding protein-3
JNK: c-Jun N-terminal kinase
MAPK: Mitogen-activated protein kinase
MCM4: Minichromosome maintenance protein-4
MDA: Malondialdehyde
MDH: Magnesium dihydroxide
MMP: Matrix metalloproteinase
mTOR: Mammalian target of rapamycin
NF-κB: Nuclear factor kappa-B
NSCLC: Non-small-cell lung cancer
OGG1: 8-Oxoguanine DNA Glycosylase 1
PBMC: Peripheral Blood Mononuclear Cells
PBS: Phosphate-buffered saline
PDH: pyruvate dehydrogenase
PDHE1α(S232): Pyruvate Dehydrogenase E1 Alpha Subunit, Serine 232
PDTC: Pyrrolidine Dithiocarbamate
PFK: phosphofructokinase
PhA: Phase angle
PI3K: Phosphoinositide-3-kinase
PKM2: Pyruvate kinase M2
RB: Retinoblastoma
RES: Resveratrol
Res-SLN: Resveratrol-loaded SLN
ROS: Reactive oxygen species
SBECD: Sulfobutylether-β-cyclodextrin
SHBG: Sex hormone-binding globulin
SIRT1: Sirtuin-1
SLN: Solid lipid nanoparticles
Sp1: Specificity Protein 1
SRB: Sulforhodamine-B assay
STAT: Signal transducer and activator of transcription
SULTs: sulfotransferases
Tf-RES-L: Transferrin-liposomal resveratrol
Tf-RES-L: Transferrin resveratrol loaded liposome/nanoparticle
TGF-β: Transforming growth factor-β
TLMA: Telomere length maintenance activity
TNF-α: Tumor Necrosis Factor α
TRAP: Telomeric repeat amplification protocol
TSP-1: Thrombospondin-1
UGTs: UDP-glucuronosyltransferases
VEGF: Vascular endothelial growth factor
XRCC1: X-Ray Repair Cross-Complementing Protein 1
